# Hydroboration of Alkynes Catalyzed by Fe(II) PCP Pincer Complexes

**DOI:** 10.1021/acs.organomet.6c00268

**Published:** 2026-09-14

**Authors:** Matthias Käfer, Luis F. Veiros, Karl Kirchner

**Affiliations:** † Institute of Applied Synthetic Chemistry, TU Wien, Getreidemarkt 9/163-AC, A-1060 Wien, Austria; ‡ Centro de Química Estrutural, Institute of Molecular Sciences, Departamento de Engenharia Química, Instituto Superior Técnico, 72971 Universidade de Lisboa, Av. Rovisco Pais, 1049 001 Lisboa, Portugal

## Abstract

A stereo- and regioselective Fe­(II)-catalyzed hydroboration of both terminal and internal alkynes with pinacolborane (HBpin) is described. The hydroboration reaction is in all cases highly *syn*-selective. The reaction requires no additives and proceeds with a catalyst loading of 1–2 mol % at 45–55 °C. An active pre-catalyst is the bench-stable alkyl Fe­(II) complex [Fe­(PCP^CH2^-*i*Pr)­(CH_2_CH_2_CH_3_)­(CO)_2_]. This complex reacts with an excess of HBpin to form the Fe­(II) pinacolborane complex [Fe­(PCP^CH2^-*i*Pr)­(κ^2^-H_2_BPin)­(CO)], which is an equally active pre-catalyst but more difficult to handle, as it decomposes in the absence of substrates. Both pre-catalysts afford the highly reactive hydride catalyst [Fe­(PCP^CH2^-*i*Pr)­(H)­(CO)] under catalytic conditions. A broad variety of aromatic and aliphatic alkynes were efficiently and selectively borylated. Mechanistic insights are provided based on experimental data and DFT calculations.

## Graphical abstract

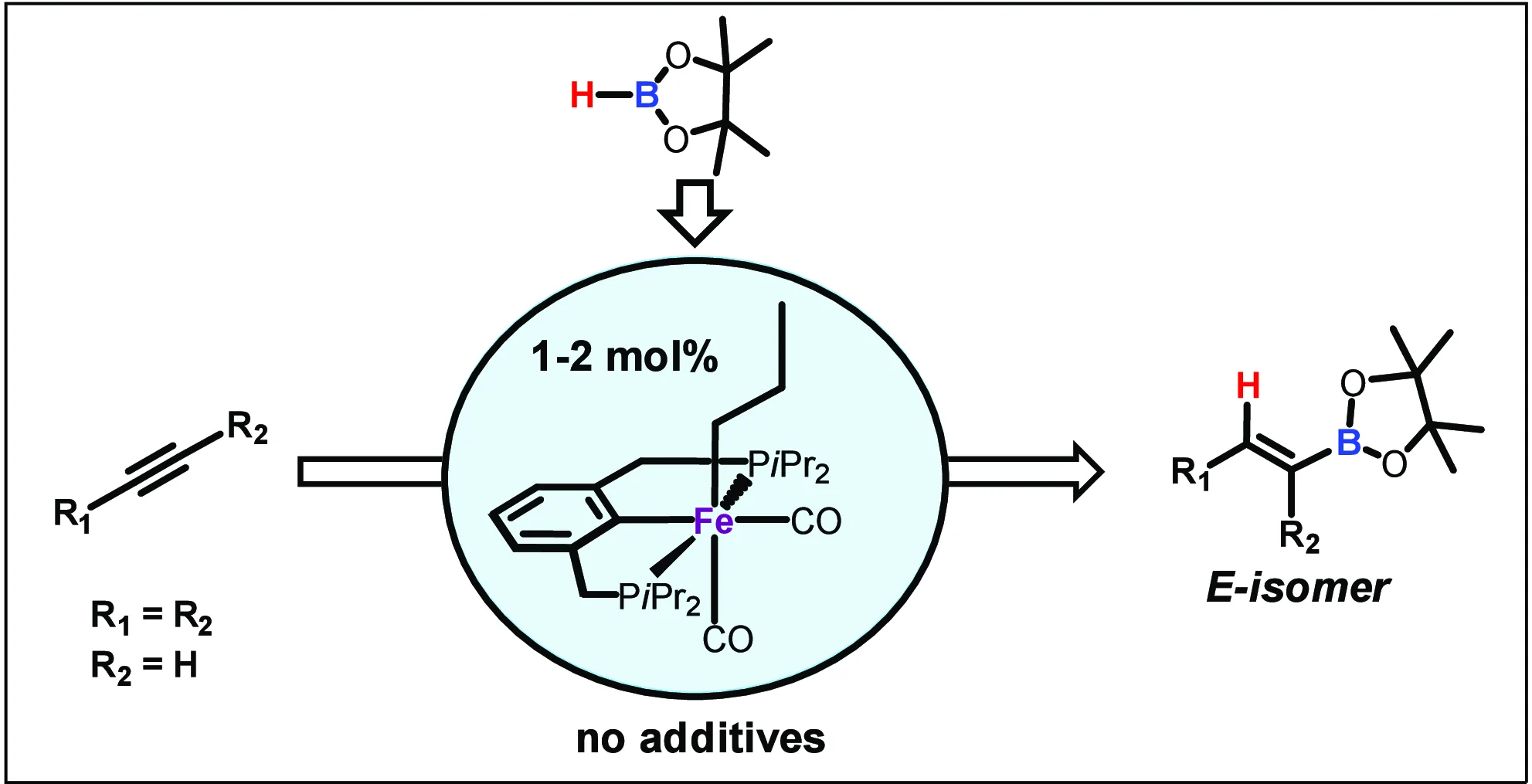

The use of organoboron reagents especially in the field of cross-coupling chemistry has gained importance within the last few decades, in part due to the emergence of hydroboration catalysts, which make these compounds easily accessible.^1,2^ In this context, the use of pinacolborane (HBpin) was introduced due to the stability of this borane and the hydroborated products.^3^ In the last years, not only precious but also nonprecious metal catalysts based on for example Mn,^4^ Co,^5^ and Ni^6^ were successfully applied for this reaction. Also, in the case of Fe several examples of the hydroboration of alkynes were reported in recent years.^7^ The addition of the borane is typically *syn* with respect to the C≡C bond, and the product selectivity, with a few exceptions,^7f,7g^ typically favors the more stable *E*-vinlyboronate (where the E/Z stereochemistry refers to the position of the borane group).

Here, we describe the activity of a series of Fe­(II) PCP pincer carbonyl complexes including the bench-stable alkyl complex [Fe­(PCP^CH2^-*i*Pr)­(CH_2_CH_2_CH_3_)­(CO)_2_][Bibr ref8] as precatalysts for the stereo- and regioselective hydroboration of terminal and internal alkynes (Scheme 1). A plausible reaction mechanism based on detailed experimental and theoretical studies is presented.

**Scheme 1:**
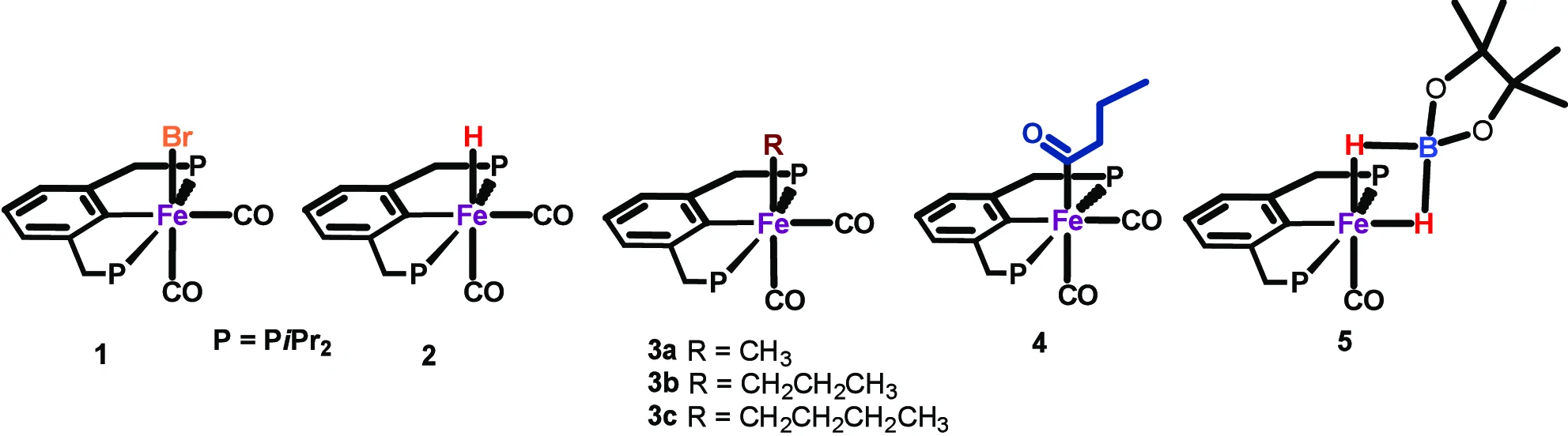
Iron­(II) PCP Pincer Complexes as Potential Catalysts for the Hydroboration of Alkynes

The catalytic performance of the known Fe­(II) complexes **1**–**5** was investigated for the hydroboration of diphenylacetylene as a model substrate. All optimization experiments are provided in the SI (Table S1). Complexes **3b** and **5** turned out to be the most active precatalysts with a catalyst loading of 1 mol % in benzene at 45 °C affording the vinyl boronic ester **S1** in essentially quantitative yield with an *E*:*Z* ratio of 99:1 (see SI, Table S1, entries 4 and 7).

Having established the optimal reaction conditions, the scope and limitations were examined as shown in Table 1. In this context, a variety of aromatic and aliphatic alkynes with both electron-withdrawing and electron-donating moieties were tested. The utilization of most *meta*- and *para*-substituted internal alkynes with electron-donating and electron-withdrawing substituents resulted in the formation of boronic esters in excellent yields and high *E*:*Z* ratios of up to 99:1 (Tables 1, S1–S4, S7–S12). Differences arose when methoxy groups were present such as in the case of 1,2-bis­(4-methoxyphenyl)­ethyne, where a lower of yield of 67% was observed (Tables 1,S10). Sterically more demanding substrates bearing substituents in the *ortho* positions such as 1,2-di-*o*-tolylethyne led to a significantly reduced yield of 40% (Tables 1, S11). Aliphatic substrates were also converted into vinyl boronic esters but required a slightly higher reaction temperature of 55 °C to achieve good yields. For instance, with 4-octyne the boronic ester **S13** was isolated in 70% yield. Thienyl moieties were also tolerated, and 1,2-di­(thien-3-yl)­ethyne was converted to **S12** in 71% yield with an *E:Z* ratio of 99:1. Moreover, terminal alkynes were also converted into vinyl boronic esters but generally required a higher catalyst loading of 2 mol % and a reaction temperature of 55 °C to achieve reasonably high yields (Tables 1, S14–S18). Gratifyingly, the boronic esters were obtained predominantly as the *E* -isomers.

**Table 1:**
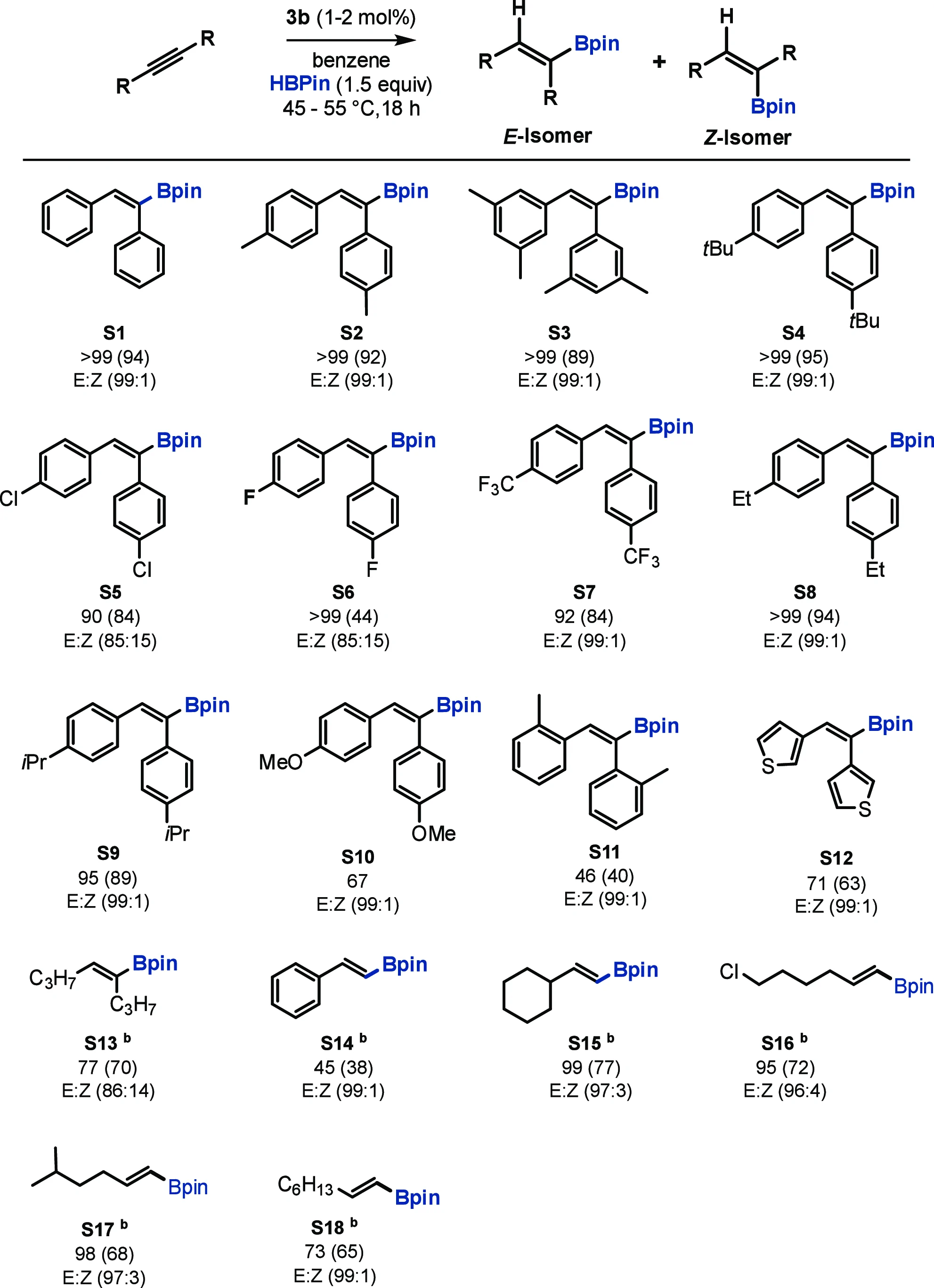
Hydroboration of Alkynes Catalyzed by **3b** ^a^

a Reaction conditions: substrate (0.5 mmol, 1 equiv), HBpin (0.75 mmol, 1.5 equiv), benzene (0.5 mL), **3b** (1 mol %), 18 h, 45 °C.

b Reaction conditions: **3b** (2 mol %), 55 °C. Educt conversions are reported with isolated yields given in parentheses. *E*:*Z* ratios have been determined by GC-MS with *n*-butyl benzene as internal standard and NMR spectroscopy.

In order to get some mechanistic insights, several experiments were carried out employing phenylacetylene as substrate under standard reaction conditions (see SI, Table S2). The homogeneity of the system was proven upon addition of one drop of Hg, which did not lead to a loss of productivity. Likewise, the presence of NEt_3_ and PPh_3_, respectively, did not affect the catalytic reaction, with conversions of 98% and 93%. In the case of the latter, however, the *E*/*Z* ratio dropped to 64:36. On the other hand, addition of 1 equiv of CN*t*Bu and P­(OMe)_3_, respectively, resulted in no or only 5% conversion. This indicates that the reaction proceeds via an inner-sphere pathway, since these strongly coordinating ligands block a vacant side for incoming substrates.

Furthermore, deuterium labeling studies were carried out with phenylacetylene-*d*_1_ and octyne-*d*_1_ as substrates (Scheme 2). In the case of phenylacetylene-*d*_1_, upon hydroboration the deuterium remained in the terminal position with 86% but also ended up with 14% at the benzylic position. In contrast, with octyne-*d*_1_ no deuterium migration was observed. These findings reveal that one predominant mechanism is operating where C–D (C–H) bond cleavage does not occur. In the case of aromatic alkynes, as observed also previously, due the higher acidity of the C–D (C–H) bond^4e^ to a small extent an alternative pathway involving C–D bond cleavage occurs. This behavior may be attributed to the different acidity of the terminal C–H bond (p*K*_a_ (aromatic) ≈ 23, p*K*_a_ (aliphatic) ≈ 25).^9^

**Scheme 2:**
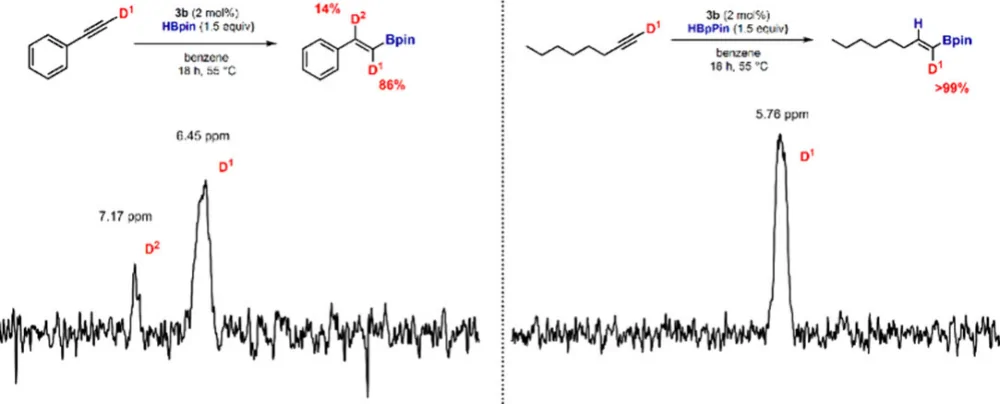
Hydroboration of Phenylacetylene-*d*_1_ and Octyne-*d*_1_ with ^2^H NMR Spectra of the Respective Products (in Benzene)

For the elucidation of a reasonable mechanism that corroborates the experimental results discussed, DFT calculations were performed.^10^ We choose pre-catalyst [Fe­(PCP^CH2^-*i*Pr)­(κ^2^-H_2_BPin)­(CO)] (**5**)^8^ as a starting point, as this complex is readily formed from [Fe­(PCP^CH2^-*i*Pr)­(CH_2_CH_2_CH_3_)­(CO)_2_] (**3b**) in the presence of an excess of HBpin under catalytic conditions. HC≡CPh was chosen as model substrate. The detailed free energy profiles obtained are provided in the SI (Figures S1 and S2), while a simplified catalytic cycle is depicted in Figure 1 with only key intermediates shown.

**Figure 1:**
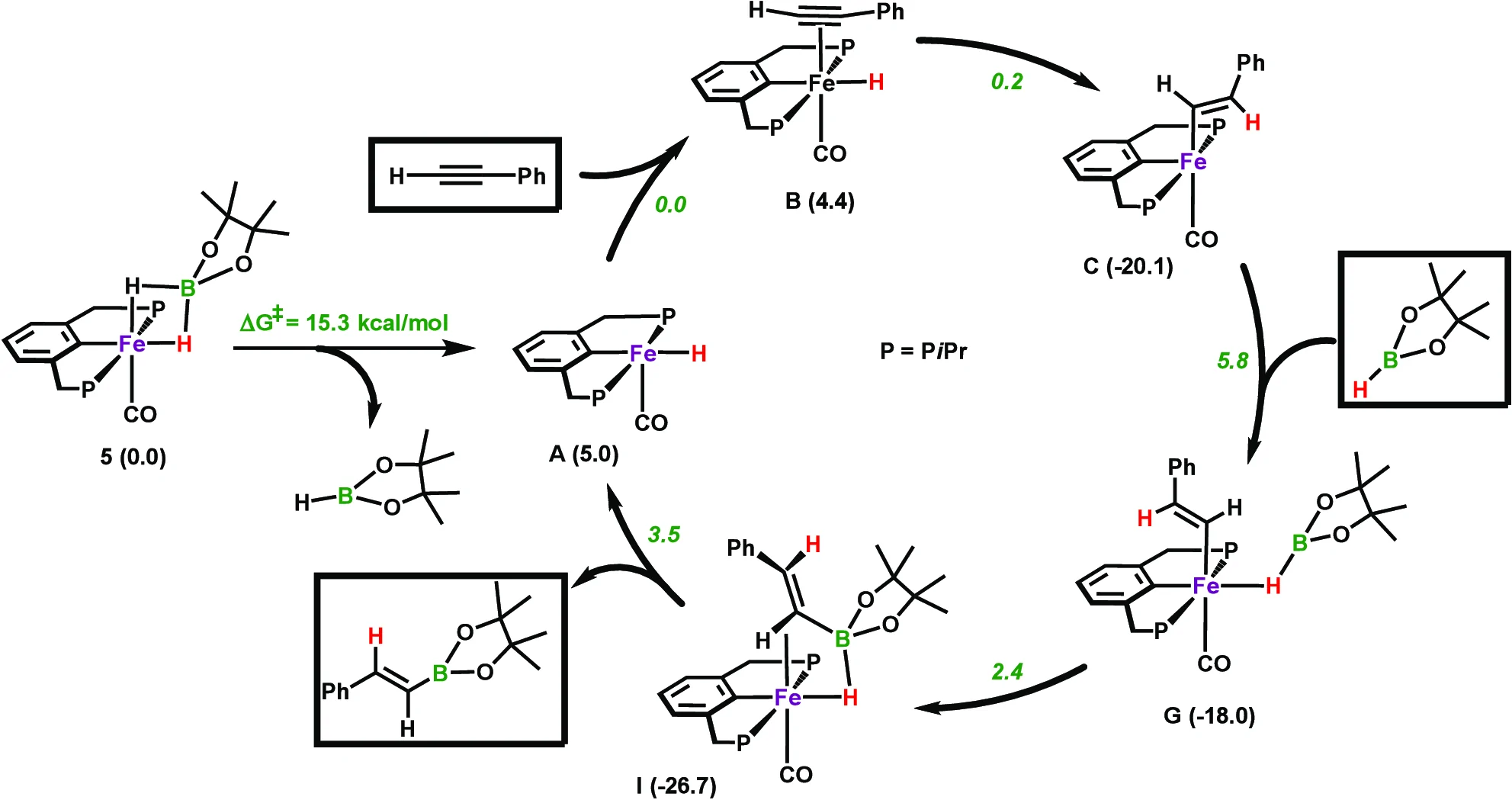
Simplified catalytic cycle for the hydroboration of terminal alkynes.

The experimental data suggest clearly that the hydroboration takes place predominantly via one distinct mechanism. For the formation of *E*-alkenylboronate esters from alkynes, catalyst initiation starts with the dissociation of HBpin from **5** to form the 16e hydride catalyst [Fe­(PCP^CH2^-*i*Pr)­(H)­(CO)] (**A**). The barrier for the dissociation of HBpin is 15.3 kcal/mol as described recently.^8^ Addition of HC≡CPh to **A** affords the η^2^-alkyne intermediate **B**, which undergoes insertion into the Fe–H bond to yield vinyl species **C**. This step is favorable from a thermodynamic point of view with Δ*G* = −24.5 kcal/mol and exhibits a negligible barrier of 0.2 kcal/mol. Addition of HBpin to **C** leads to the formation of the van der Waals adduct **D**, which then reacts to give the vinyl HBpin intermediate **E**, which rearranges to **F** and then to **G**, which differs only by the orientation of the coordinated HBpin ligand. The three conformers have similar stability within 4.4 kcal/mol, and the highest barrier for these rotational changes is Δ*G* ^‡^ = 5.8 kcal/mol measured from intermediate **E** to **TS**_**FG**_ via **F**, which is also the highest barrier along the cycle. Subsequently, the κ^1^-bound HBpin molecule develops a carbon–boron bond to yield the vinyl boronate complex **H**. This process is exergonic by 6 kcal/mol with a small barrier of 2.4 kcal/mol. At this stage, both the B–H and the Fe–H bonds are almost unchanged (1.38 and 1.59 Å, respectively). Along the further reaction pathway from **H** to **I** and **J** the B–H bond gets gradually cleaved through transition state **TS**_**IJ**_ (Δ*G* ^‡^ = 3.1 kcal/mol) to give finally complex **J**, where the vinyl boronate product is completely formed and only loosely bound. After product release, the hydride intermediate **A** is reformed, initiating the next catalytic cycle. Overall, the process is favorable, from the thermodynamic point of view, with Δ*G* = −35.0 kcal/mol, from **A** to **J**. It is important to note that this transformation requires no C–H bond activation of the alkyne to give an acetylide intermediate as found recently for a manganese-catalyzed hydroboration of alkynes in the case of aromatic alkynes.^4e^

In conclusion, the bench-stable alkyl Fe­(II) complex turned out to be an efficient catalyst for the additive-free stereo- and regioselective hydroboration of terminal alkynes with HBpin. Hydroboration takes place with a catalyst loading of 1–2 mol % at 45–55 °C with a high *E*-selectivity. The catalytic process is initiated by the dissociation of HBpin from complex **5** (this complex is readily formed from [Fe­(PCP^CH2^-*i*Pr)­(CH_2_CH_2_CH_3_)­(CO)_2_] (**3b**) in the presence of an excess of HBpin as under catalytic conditions), thereby forming the catalytically active 16e^–^ Fe­(II) hydride species [Fe­(PCP^CH2^-*i*Pr)­(H)­(CO)] (**A**). A broad variety of aromatic and aliphatic alkynes were efficiently and selectively borylated. Mechanistic insights are provided based on experimental and computational studies.

## Supplementary Material





## Notes

Cartesian coordinates for DFT-optimized structures (XYZ)
^1^H, ^13^C­{^1^H}, and ^31^P­{H} NMR spectra of all compounds and complete computational details and corresponding references (PDF)
