# Supplementary material for: Hydroboration of Alkynes Catalyzed by Fe(II) PCP Pincer Complexes
Source: Organometallics. 2026 Sep 14;45(18):2195–8. doi: 10.1021/acs.organomet.6c00268 (PMC13626059; doi:10.1021/acs.organomet.6c00268)
Supplement: Supplementary file 1 [file om6c00268_si_002.pdf]

Supporting Information

Hydroboration of Alkynes Catalyzed by Fe(II) PCP Pincer Complexes

Matthias Käfer,[†] Luis F. Veiros,[‡] Karl Kirchner*,[†]

E-mail: karl.kirchner@tuwien.ac.at

[†] Institute of Applied Synthetic Chemistry, TU Wien, Getreidemarkt 9/163-AC, A-1060 Wien, Austria.
[‡] Centro de Química Estrutural, Institute of Molecular Sciences, Departamento de Engenharia Química, Instituto Superior Técnico, Universidade de Lisboa, Av. Rovisco Pais, 1049 001 Lisboa, Portugal

Table of Content

Experimental Section S2
Computational Details S5
Spectroscopic Data of Organic Compounds S7
References S13
NMR Spectra of New Organic Products S17

Experimental Section

General Information. All manipulations were performed under an inert atmosphere of argon by using Schlenk techniques or in an MBraun inert-gas glovebox. The solvents were purified according to standard procedures.[1] The deuterated solvents were purchased from Eurisotop SAS and dried over 4 Å molecular sieves. Most starting materials are known compounds and were used as obtained from commercial resources. Complexes [Fe(PCP^{CH2}-*i*Pr)(CO)$_2$Br] (**1**),[2] [Fe(PCP^{CH2}-*i*Pr)(H)(CO)$_2$] (**2**), [Fe(PCP^{CH2}-*i*Pr)(CH$_3$)(CO)$_2$] (**3a**), [Fe(PCP^{CH2}-*i*Pr)(CH$_2$CH$_2$CH$_3$)(CO)$_2$] (**3b**), [Fe(PCP^{CH2}-*i*Pr)(CH$_2$CH$_2$CH$_2$CH$_3$)(CO)$_2$] (**3b**), [Fe(PCP^{CH2}-*i*Pr)((C=O)-CH$_2$CH$_2$CH$_3$)(CO)$_2$] (**4**) and [Fe(PCP^{CH2}-*i*Pr)(κ^2-H$_2$BPin)(CO)] (**5**)[3] were prepared according to the literature. Organic substrates were prepared according to modified literature procedures.[4,5]

^{1}H, ^{13}C{^{1}H} and ^{31}P{^{1}H} NMR spectra were recorded on Bruker AVANCE-250, AVANCE-400 and AVANCE-600 spectrometers. ^{1}H and ^{13}C{^{1}H} NMR spectra were referenced internally to residual protio-solvent and solvent resonances, respectively, and are reported relative to tetramethylsilane (δ = 0 ppm). ^{31}P{^{1}H} NMR spectra were referenced externally to H_3PO_4 (85%) (δ = 0 ppm).

Infrared spectra were recorded in attenuated total reflection (ATR) mode on a PerkinElmer Spectrum Two FT-IR spectrometer or on a Bruker Vertex 80 FT-IR spectrometer. GC-MS analyses were conducted on a ISQ LT Single quadrupole MS (Thermo Fisher) directly interfaced to a TRACE 1300 Gas Chromatographic system (Thermo Fisher), using a Rxi-5Sil MS (30 m, 0.25 mm ID) cross-bonded dimethyl polysiloxane capillary column at a carrier flow of He 1.5 mL/min.

High resolution-accurate mass spectra were recorded on a hybrid Maxis Qq-aoTOF mass spectrometer (Bruker Daltonics, Bremen, Germany) fitted with an ESI source or an Agilent 6545 QTOF mass spectrometer equipped with an Agilent Dual AJS ESI ion source (Agilent Technologies, Santa Clara, CA, USA). Measured accurate mass data of the [M]+ ions for confirming calculated elemental compositions were typically within ±5 ppm accuracy. The mass calibration was done with a commercial mixture of perfluorinated trialkyl-triazines (ES Tuning Mix, Agilent Technologies, Santa Clara, CA, USA).

General Procedure for Optimization Reactions (Data depicted in Table S1). A screw cap vial (8 mL) was charged with the respective pre-catalyst (mol%), diphenylacetylene (0.10 mmol, 1 equiv.), HBpin (0.15 mmol, 1.5 equiv.) and filled with benzene (0.5 mL) inside an argon filled glovebox. The pre-catalyst was added last as a stock solution in benzene. The vial was closed, transferred out of the glovebox and was stirred for the given reaction time at a given temperature. After the reaction, the vial was cooled to room temperature and exposed to air to decompose the catalyst. A sample (2 µL) was taken and was analysed via GC-MS.

Table S1. Optimization Reactions for the Hydroboration of Diphenylacetylene.[a]

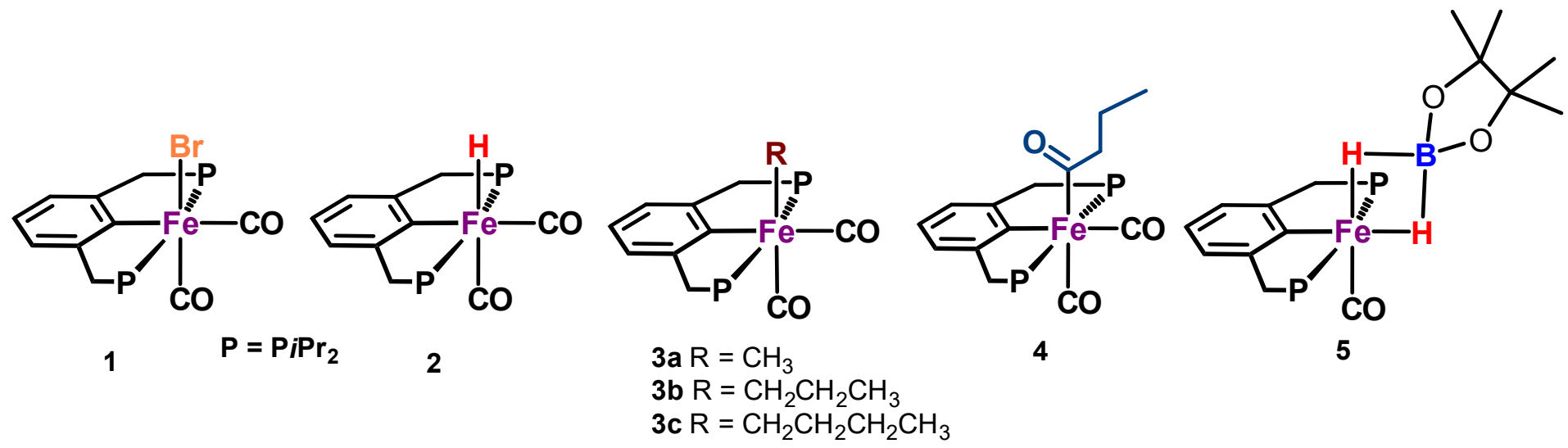

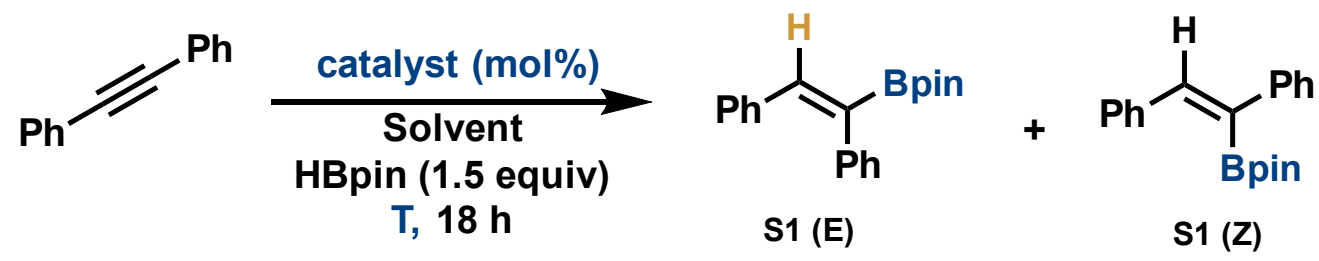

Entry	Catalyst (mol%)	T (°C)	Solvent	E/Z ratio	Conversion
1	**1** (3)	45	Benzene		
9	**2** (1)	45	Benzene		
3	**3a** (1)	45	Benzene	94:6	68
**4**	**3b (1)**	**45**	**Benzene**	**99:1**	**>99**
5	**3c** (1)	45	Benzene	95:5	93
6	**4** (1)	45	Benzene		
**7**	**5 (1)**	**45**	**Benzene**	**99:1**	**>99**
8	**3b** (1)	25	Benzene	99:1	68
9	**3b** (1)	45	THF	99:1	79
10	**3b** (1)	45	Toluene	99:1	85
11	**3b** (2)	45	DCM[b]		25
12	**3b** (2)	45	MeCN[b]		45

[a]Reaction conditions. 0.1 mmol (1 equiv.) substrate, x mol% catalyst, 0.5 mL solvent, HBpin (1.5 equiv., 0.15 mmol), temperature, 18 h, conversions and E/Z ratios are reported (determined by GC-MS; *n*-butyl benzene as internal standard). [b]48 h.

Complexes **3b** and **5** performed identical under the reported reaction conditions. Therefore, **3b** was utilized as pre-catalyst since this compound is bench stable while **5** rapidly decomposes in the absence of HBpin as already reported recently.[3] Complex **5** is readily formed in the presence of an excess of HBpin.

General Procedure for Hydroboration of Alkynes with 3b as Catalyst. A screw cap vial (8mL) was charged with **3b** (1-2 mol%), the corresponding alkyne (0.5 mmol, 1 equiv.), HBpin (0.75 mmol, 1.5 equiv.) and dissolved in benzene (1 mL) inside an argon filled glovebox. The complex was added last, as a stock solution in benzene. The vial was closed, transferred out of the glovebox and was stirred for 18 h at 45°C if not stated otherwise. After the reaction, the vial was cooled to r.t., exposed to air to inactive the catalyst. A 2µL sample was taken and was analysed via GC-MS. Water (2 mL) was added to the remaining solution. The phases were separated, followed by extracting the aqueous phase with Et_2O (5 mL). The combined organic phases were filtered through a plug of silica, followed by evaporation of the solvent. The crude products were purified via column chromatography, if necessary. (silica gel, PE:EE 50-20:1, depending on the product, or *n*-pentane: Et_2O 50-20:1, for volatile products.

General Procedure for Poisoning Experiments. Poisoning with NEt_3. A screw cap vial (8mL) was charged diphenylacetylene (36.2 mg, 0.2 mmol, 1 equiv.), HBpin (44.2 µL, 0.3mmol, 1.5 equiv.) dissolved in benzene (1mL), and **3b** as a stock solution in benzene (1 mg, 2.03 µmol, 1 mol%) inside an argon filled glovebox. NEt_3 (28 µL, 0.2 mmol, 1 equiv.) was added. The vial was closed, transferred out of the glovebox and was stirred for 18 h at 45°C. After completion of the reaction time, the vial was opened to quench the catalyst, and a 2µL sample was taken and subjected to GC-MS analysis.

Poisoning with $P(OMe)_3$. A screw cap vial (8mL) was charged diphenylacetylene (36.2 mg, 0.2 mmol, 1 equiv.), HBpin (44.2 µL, 0.3 mmol, 1.5 equiv.) dissolved in benzene (1mL), and **3b** as a stock solution in benzene (1 mg, 2.0 µmol, 1 mol%) inside an argon filled glovebox. $P(OMe)_3$ (24 µL, 0.2 mmol, 1 equiv.) was added. The vial was closed, transferred out of the glovebox and was stirred for 18 h at 45°C. After completion of the reaction time, the vial was opened to quench the catalyst, and a 2 µL sample was taken and subjected to GC-MS analysis.

Poisoning with PPh_3. A screw cap vial (8mL) was charged diphenylacetylene (36.2 mg, 0.2 mmol, 1 equiv.), HBpin (44.2 µL, 0.3mmol, 1.5 equiv.) dissolved in benzene (1mL), and **3b** as a stock solution in benzene (1 mg, 2.0 µmol, 1 mol%) inside an argon filled glovebox. PPh_3 (53.3 mg, 0.2 mmol, 1 equiv.) was added. The vial was closed, transferred out of the glovebox and was stirred for 18 h at 45°C. After completion of the reaction time, the vial was opened to quench the catalyst, and a 2 µL sample was taken and subjected to GC-MS analysis.

Poisoning with CN*t*Bu. A screw cap vial (8mL) was charged diphenylacetylene (36.2 mg, 0.2 mmol, 1 equiv.), HBpin (44.2 µL, 0.3mmol, 1.5 equiv.) dissolved in benzene (1mL), and **3b** as a stock solution in benzene (1 mg, 2.0 µmol, 1 mol%) inside an argon filled glovebox. CN*t*Bu (21 µL, 0.2 mmol, 1 equiv.) was added. The vial was closed, transferred out of the glovebox and was stirred for 18 h at 45°C. After completion of the reaction time, the vial was opened to quench the catalyst, and a 2µL sample was taken and subjected to GC-MS analysis.

Poisoning with Mercury. A screw cap vial (8mL) was charged diphenylacetylene (36.2 mg, 0.2 mmol, 1 equiv.), HBpin (44.2 µL, 0.3mmol, 1.5 equiv.) dissolved in benzene (1mL), and **3b** as a stock solution in benzene (1 mg, 2.0 µmol, 1 mol%) inside an argon filled glovebox. 2 drops of mercury were added. The vial was closed, transferred out of the glovebox and was stirred for 18 h at 45°C. After completion of the reaction time, the vial was opened to quench the catalyst, and a 2µL sample was taken and subjected to GC-MS analysis.

Table S2. Hydroboration of Diphenylacetylene with HBpin in the Presence of Additives.

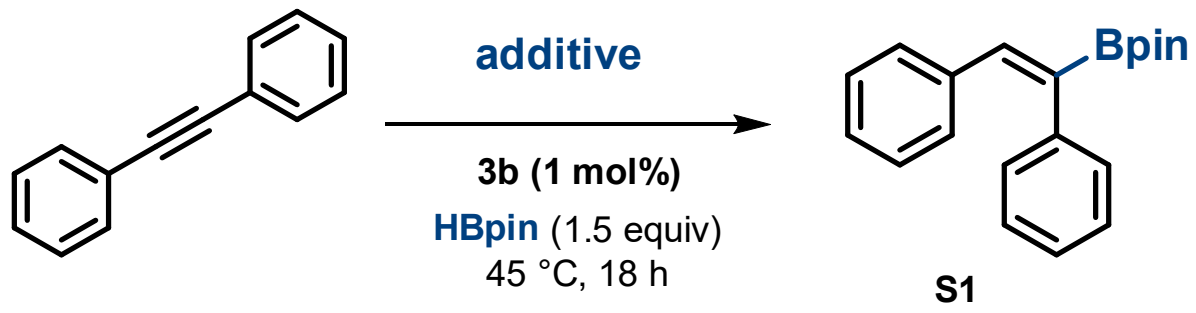

Entry	Additive	*E/Z* ratio	Conversion[a]
1	NEt_3	99:1	98
2	$P(OMe)_3$	99:1	5
3	PPh_3	64:36	93[b]
4	CNtBu		<1
5	mercury	98:2	97

[a] Conversions and *E/Z* ratio determined by GC/MS an, referenced against *n*-butyl benzene as internal standard. [b] Stilbene formation observed (16%)

Deuterium Labeling Studies. Phenylacetylene-d_1. A screw cap vial (8mL) was charged phenylacetylene-d_1(22.5 µL mg, 0.2 mmol, 1 equiv.), HBpin (44.2 µL, 0.3 mmol, 1.5 equiv.) dissolved in benzene (1mL), and **3b** was added as a stock solution in benzene (2 mg, 4.1 µmol, 2 mol%) inside an argon filled glovebox. The vial was closed, transferred out of the glovebox and was stirred for 18 h at 55°C. After completion of the reaction time, the vial was opened to quench the catalyst, and the solution was filtered into an NMR tube and subjected to ^{2}H NMR spectroscopy.

Octyne-d_1. A screw cap vial (8mL) was charged octyne-d_1 (30.0 µL mg, 0.2 mmol, 1 equiv.), HBpin (44.2 µL, 0.3 mmol, 1.5 equiv.) dissolved in benzene (1mL), and **3b** was added as a stock solution in benzene (2 mg, 4.1 µmol, 2 mol%) inside an argon filled glovebox. The vial was closed, transferred out of the glovebox and was stirred for 18 h at 55°C. After completion of the reaction time, the vial was opened to quench the catalyst, and the solution was filtered into an NMR tube and subjected to ^{2}H NMR spectroscopy.

Computational Details. Calculations were performed using the GAUSSIAN 09 software package[5] and the PBE0 functional, without symmetry constraints. That functional uses a hybrid generalized gradient approximation (GGA), including 25 % mixture of Hartree-Fock[6] exchange with DFT[7] exchange-correlation, given by Perdew, Burke and Ernzerhof functional (PBE).[8] The optimized geometries were obtained with the Stuttgart Effective Core Potentials and associated basis set (SDD)[9] for Fe, and a standard 6-31G(d,p)[10] for the remaining elements (basis b1). Transition state optimizations were performed with the Synchronous Transit-Guided Quasi-Newton Method (STQN) developed by Schlegel *et al*,[11] following extensive searches of the Potential Energy Surface. Frequency calculations were performed to confirm the nature of the stationary points, yielding one imaginary frequency for the transition states and none for the minima. Each transition state was further confirmed by following its vibrational mode downhill on both sides and obtaining the minima presented on the energy profile. The electronic energies (E_{b1}) obtained at the PBE0/b1 level of theory were converted to free energy at 298.15 K and 1 atm (G_{b1}) by using zero-point energy and thermal energy corrections based on structural and vibration frequency data calculated at the same level.

Single point energy calculations were performed on the geometries obtained at the PBE0/b1 level using a 6-311++G(d,p) basis set[12] (basis b2). Solvent effects (benzene) were accounted for in all calculations (including geometry optimizations) by means of the Polarisable Continuum Model (PCM) initially devised by Tomasi and coworkers[13] with radii and non-electrostatic terms of the SMD solvation model, developed by Truhler *et al*.[14] The free energy values presented (G_{b2}-D3) were corrected for dispersion by means of Grimme DFT-D3 method[15] with Becke and Johnson short distance damping,[16] being derived from the electronic energy values obtained at the PBE0-D3/b2//PBE0/b1 level (E_{b2}-D3) according to the following expression: $(G_{b2}\text{-D3}) = (E_{b2}\text{-D3}) + G_{b1} - E_{b1}$

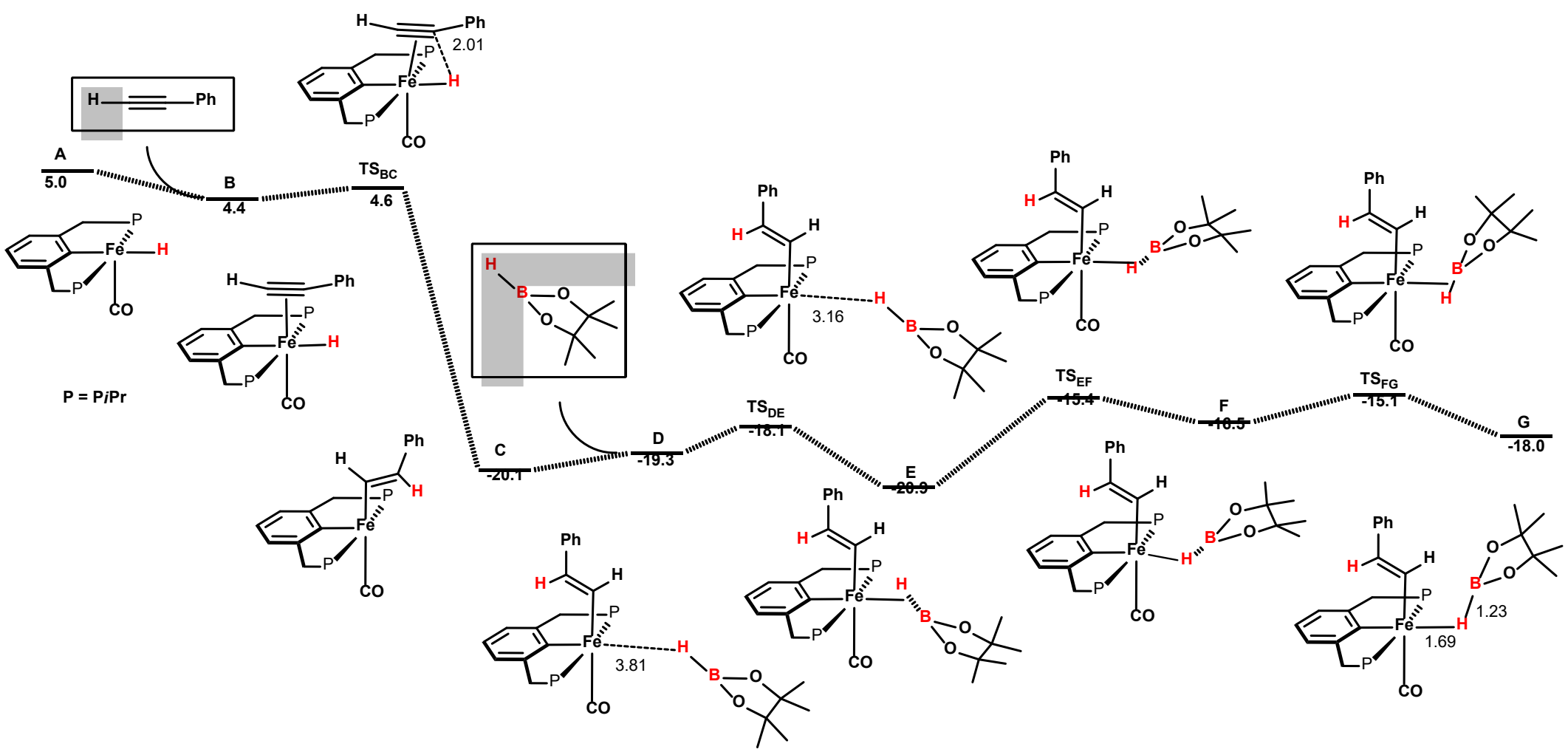

Figure S1. Free energy profile of hydroboration of HC≡CPh. Free energies (kcal/mol) are referred to [Fe(PCP^{CH2})(κ^2*H,H*-H_2Bpin)(CO] (**5**).

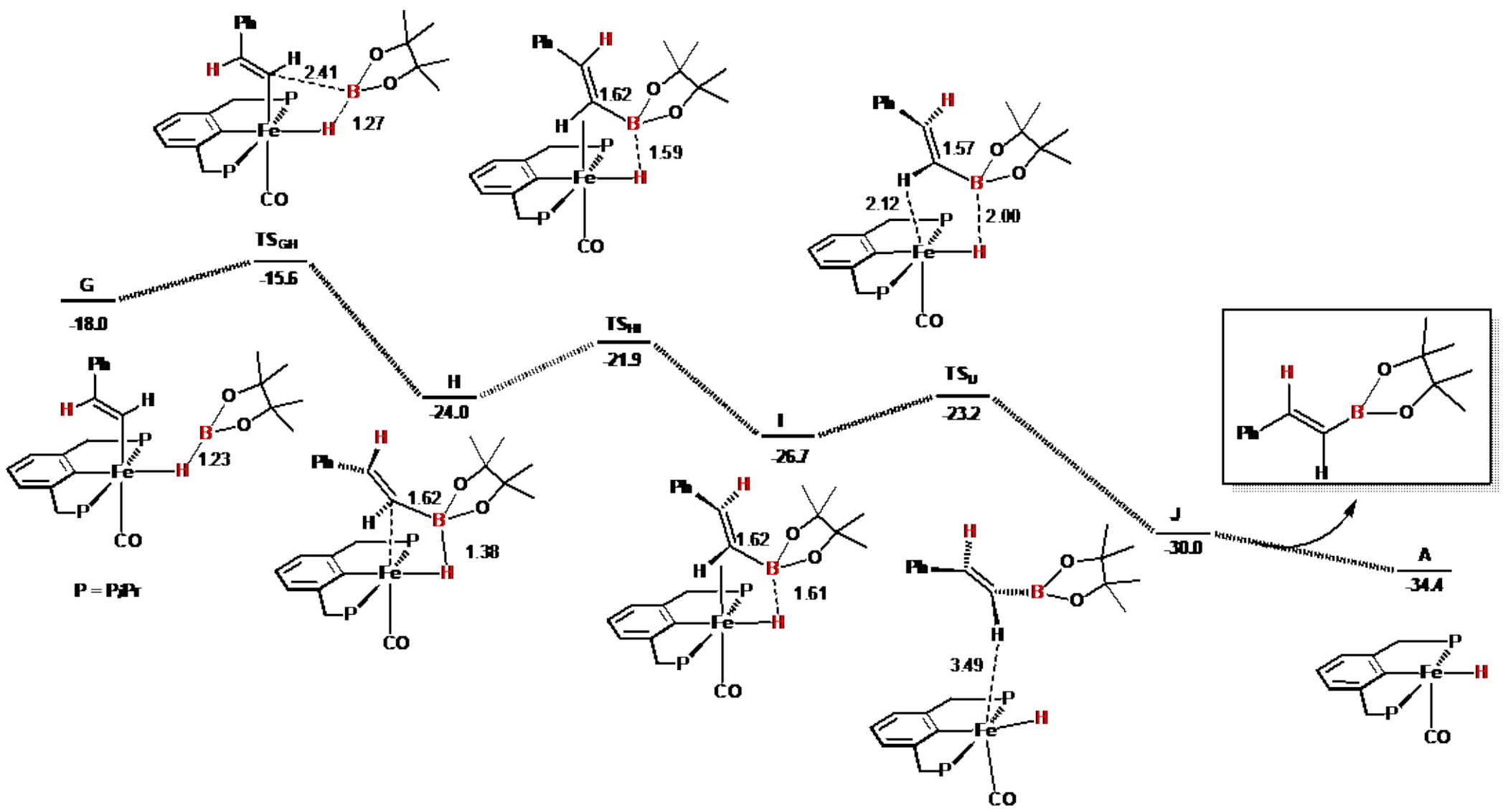

Figure S2. Free energy profile of hydroboration of HC≡CPh. Free energies (kcal/mol) are referred to [Fe(PCP^{CH2})(κ^2*H,H*-H_2Bpin)(CO] (**5**).

Spectroscopic Data of Organic Compounds

(Z)-2-(1,2-diphenylvinyl)-4,4,5,5-tetramethyl-1,3,2-dioxaborolane (S1)

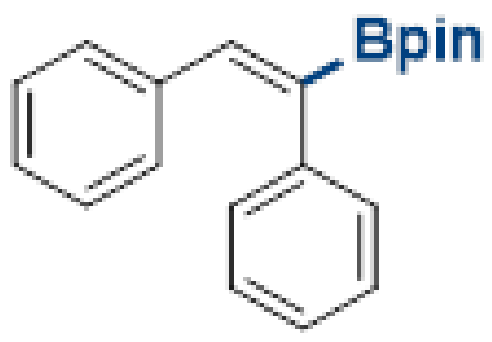

Conversion: >99% (E:Z 1:99). Yield: 143.9 mg (94% isolated yield). Rt = 8.079 min (Method B), m/z = 306.207. ^{1}H NMR (400 MHz, CD_2Cl_2, 20 °C, ppm): δ 7.36 (s, 1H, C-*H*, benzylic position), 7.31 – 7.21 (m, 5H, C-*H* arom), 7.20 – 7.09 (m, 5H, C-*H* arom), 1.31 (s, 12H, C(C*H*$_3$)$_2$). ^{13}C{^{1}H} NMR (101 MHz, CD_2Cl_2, 20 °C, ppm) δ 143.5 (*C*-H,), 141.3 (C_q), 137.4 (C_q), 130.3 (s, *C*H,), 129.2 (s, *C*H), 128.7 (s, *C*H), 128.3 (s, *C*H), 128.2 (s, *C*H), 126.6 (s, *C*H), 84.2 (s, C_q), 25.0 (s, OC(*C*H$_3$)$_2$). ^{11}B{^{1}H} NMR (128 MHz, CD_2Cl_2, 20 °C, ppm) δ 30.7 (s, B). Spectroscopic data correspond to literature data.[17]

(Z)-2-(1,2-di-p-tolylvinyl)-4,4,5,5-tetramethyl-1,3,2-dioxaborolane (S2)

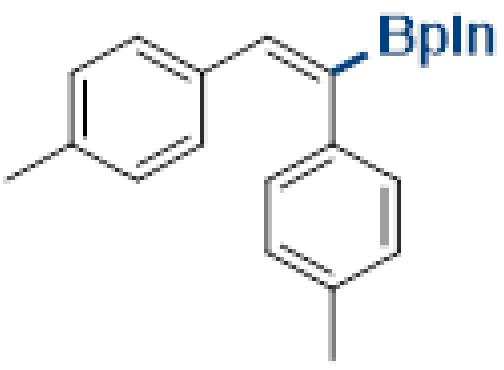

Conversion: >99% (E:Z 1:99). Yield: 153.4 mg (92% isolated yield). Rt = 8.677 min (Method B), m/z = 334.254. ^{1}H NMR (400 MHz, $CDCl_3$, 20 °C, ppm): δ 7.30 (s, 1H, C-*H*, benzylic position), 7.07 (s, 4H, C-*H*), 7.01 – 6.90 (m, 4H, C*H*), 2.33 (s, 3H, C*H*$_3$), 2.26 (s, 3H, C*H*$_3$), 1.30 (s, 12H, C*H*$_3$(Bpin)). ^{13}C{^{1}H} NMR (101 MHz, $CDCl_3$, 20 °C, ppm): δ 142.9 (s, *C*-H, benzylic) 137.6 (s, C_q – C=C), 137.4 (s, C_q), 135.6 (s, C_q-CH$_3$), 134.3 (s, C_q-CH$_3$), 129.9 (s, *C*H), 129.0 (s, *C*H), 128.7 (s, *C*H), 128.6 (s, *C*H), 128.4 (s, *C*H), 83.7 (s, C_q(BPin)), 24.8 (s, *C*H$_3$(BPin)), 21.3 (s, *C*H$_3$), 21.3 (s, *C*H$_3$). ^{11}B{^{1}H} NMR (128 MHz, $CDCl_3$, 20 °C, ppm) δ 30.7 (s, B). Spectroscopic data correspond to literature data.[17]

(Z)-2-(1,2-bis(3,5-dimethylphenyl)vinyl)-4,4,5,5-tetramethyl-1,3,2-dioxaborolane (S3)

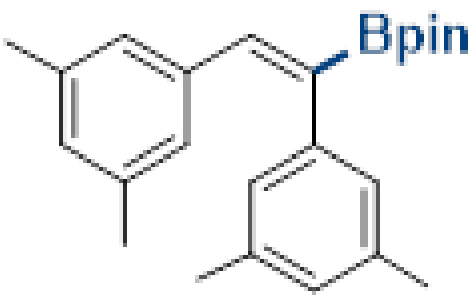

Conversion: >99% (E:Z 1:99). Yield: 161.3 mg (89% isolated yield). Rt = 8.701 min (Method B), m/z = 362.262. ^{1}H NMR (400 MHz, $CDCl_3$, 20 °C, ppm): δ 7.37 (s, 1H, C-H_p,), 7.25 (s, 1H, C-*H*, benzylic position)), 6.85 (s_{broad}, 1H, C-H_p), 6.78 (tt, *J* = 2.4, 0.8 Hz, 2H, C-H_o), 6.69 (dq, *J* = 1.8, 0.6 Hz, 2H, C-H_o), 2.25 (q, *J* = 0.7 Hz, 6H, C*H*$_3$), 2.12 (d, *J* = 0.7 Hz, 6H, C*H*$_3$), 1.30 (s, 12H, C*H*$_3$, (Bpin)).^{13}C{^{1}H} NMR (101 MHz, $CDCl_3$, 20 °C, ppm): δ 143.2 (s, *C*-H, benzylic position), 140.6 (s, *Cq*), 137.5 (s, *Cq*-CH$_3$), 137.1 (s, *Cq*-CH$_3$), 136.9(s, *Cq*), 129.4 (s, *C*H $_p$), 128.24 (s, *C*H $_o$), 128.14 (s, *C*H $_p$), 126.44 (s, *C*H $_o$), 83.7(s, C_q(BPin)), 24.9(s, *C*H$_3$(BPin)), 21.5(s, *C*H$_3$), 21.3 (s, *C*H$_3$) C_q-B not observed. ^{11}B{^{1}H} NMR (128 MHz, $CDCl_3$, 20 °C, ppm) δ 31.1 (s, B). Anal. Calcd for $C_{24}H_{31}BO_2$: C, 79.56; H, 8.62. Found: C, 79.69; H, 8.45.

(Z)-2-(1,2-bis(4-(tert-butyl)phenyl)vinyl)-4,4,5,5-tetramethyl-1,3,2-dioxaborolane (S4)

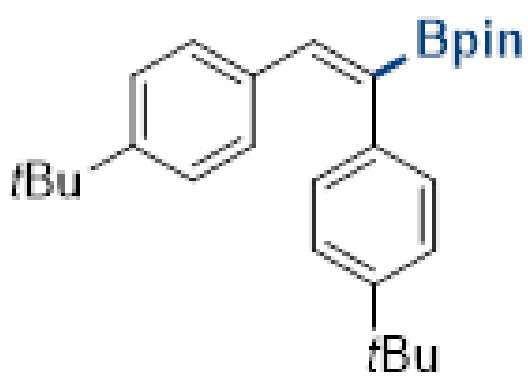

Conversion: >99% (E:Z 1:99). Yield: 198.7 mg (95% isolated yield).Rt = 10.171 min (Method B), m/z = 418.341.^{1}H NMR (400 MHz, $CDCl_3$, 20 °C, ppm): δ 7.31 – 7.29 (m, 2H, C*H*), 7.28 (d, *J* = 2.0 Hz, 1H, C*H* benzylic position), 7.14 – 7.09 (m, 4H, C*H*), 7.02 – 6.97 (m, 2H, C*H*), 1.33 (s, 9H, C(C*H*$_3$)$_3$), 1.30 (s, 12H, C*H*$_3$), 1.25 (s, 9H C(C*H*$_3$)$_3$). ^{13}C{^{1}H} NMR (101 MHz, $CDCl_3$, 20 °C, ppm): δ 150.8 (s, *Cq*C(CH$_3$)$_3$),149.0 (s, *Cq*C (CH$_3$)$_3$), 143.0 (s, *C*-H, benzylic), 137.7(s, *Cq*), 134.4 (s, *Cq*), 129.9 (s, *C*H), 128.5 (s, *C*H), 125.3 (s, *C*H), 124.9 (s, *C*H), 83.8 (s, *Cq* (Bpin)), 34.7 (s, *Cq*(CH$_3$)$_3$), 34.6 (s, *Cq*(CH$_3$)$_3$), 31.6 (s,

C($\underline{C}H_3)_3$), 31.3 (s, C($\underline{C}H_3)_3$), 24.9 (s, $\underline{C}H_3$ (BPin)).^{11}B{^{1}H} NMR (128 MHz, $CDCl_3$, 20 °C, ppm) δ 30.8 (s, B). Anal. Calcd for $C_{28}H_{39}BO_2$: C C, 80.37; H, 9.40. Found: C, 80.44; H, 9.48.

***(Z)*-2-(1,2-bis(4-chlorophenyl)vinyl)-4,4,5,5-tetramethyl-1,3,2-dioxaborolane (S5)**

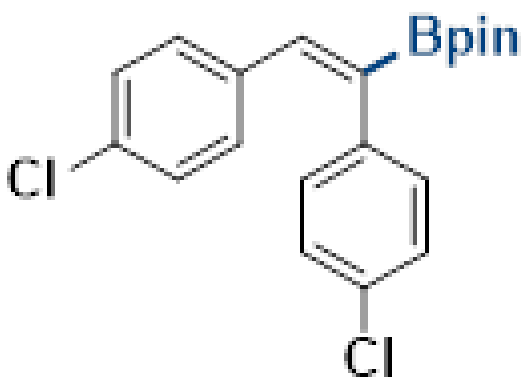

Conversion: 90% (E:Z 1:99). Yield: 157.1 mg (84% isolated yield). Rt = 9.555 min (Method B), m/z = 374.147. ^{1}H NMR (400 MHz, $CDCl_3$, 20 °C, ppm): δ 7.36 (s, 1H, benzylic position), 7.25 – 7.21 (m, 2H, C$\underline{H}$), 7.14 – 7.06 (m, 4H, C$\underline{H}$), 7.02 – 6.94 (m, 2H, C$\underline{H}$), 1.30 (s, 12H, C$\underline{H_3}$). ^{13}C{^{1}H} NMR (101 MHz, $CDCl_3$, 20 °C, ppm): δ 142.5 (s, $\underline{C}$-H, benzylic), 138.5 (s, $\underline{Cq}$), 135.2 (s, $\underline{Cq}$), 133.7 (s, $\underline{Cq}$), 132.5 (s, $\underline{Cq}$) 131.2(s, $\underline{C}$H), 130.4 (s, $\underline{C}$H), 128.8 (s, $\underline{C}$H), 128.4 (s, $\underline{C}$H), 84.2 (s, $\underline{Cq}$ (Bpin)), 24.93 (s, $\underline{C}H_3$ (BPin)). ^{11}B{^{1}H} NMR (128 MHz, $CDCl_3$, 20 °C, ppm) δ 30.7 (s, B). Spectroscopic data correspond to literature data.[18]

(*Z*)-2-(1,2-bis(4-fluorophenyl)vinyl)-4,4,5,5-tetramethyl-1,3,2-dioxaborolane (S6)

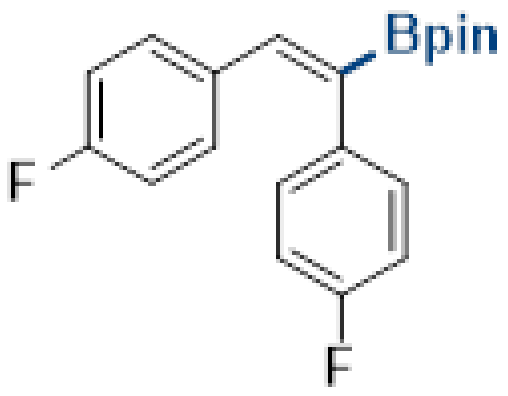

Conversion: >99% (E/Z: 4/96). Yield: 160.8 mg (94% isolated yield). Rt = 7.977 min (Method B), m/z = 342.179. ^{1}H NMR (400 MHz, $CDCl_3$, 20 °C, ppm): δ 7.22 (s, 1H, benzylic position), 7.03 – 6.98 (m, 2H, C$\underline{H}$), 6.95 – 6.89 (m, 2H, C$\underline{H}$), 6.89 – 6.82 (m, 2H, C$\underline{H}$), 6.76 – 6.69 (m, 2H, C$\underline{H}$), 1.20 (s, 12H, C$\underline{H_3}$).^{13}C{^{1}H} NMR (101 MHz, $CDCl_3$, 20 °C, ppm) δ 163.4 (d, J = 40.6 Hz, $\underline{C_q}$-F), 160.9 (d, J = 37.6 Hz, $\underline{C_q}$-F), 142.4 (s, $\underline{C}$H, benzylic position), 136.0 (d, J = 3.4 Hz, $\underline{Cq}$), 133.0 (d, J = 3.0 Hz, $\underline{Cq}$), 131.8 (d, J = 8.1 Hz, $\underline{C}$H), 130.6 (d, J = 7.8 Hz, $\underline{C}$H), 115.5 (d, J = 21.2 Hz, $\underline{C}$H), 115.1 (d, J = 21.3 Hz, $\underline{C}$H), 84.1 (s, $\underline{Cq}$ (Bpin)), 24.9 (s, $\underline{C}H_3$ (BPin)). ^{11}B{^{1}H} NMR (128 MHz, $CDCl_3$, 20 °C, ppm) δ 30.9 (s, B). Spectroscopic data correspond to literature data.[19]

***(Z)*-2-(1,2-bis(4-(trifluoromethyl)phenyl)vinyl)-4,4,5,5-tetramethyl-1,3,2-dioxaborolane (S7)**

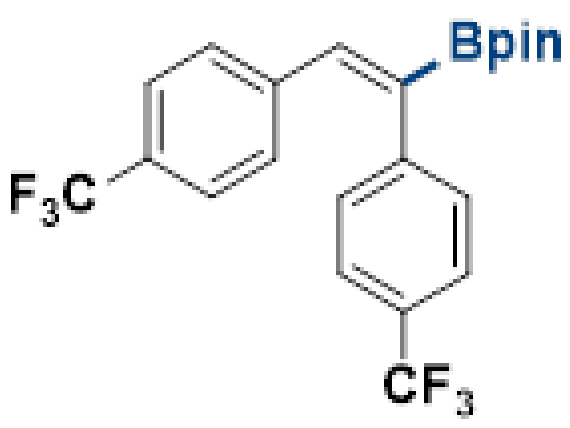

Conversion: 92% (E/Z: 1/99). Yield: 185.7 mg (84% isolated yield). Rt = 7.640 min (Method B), m/z= 442.171. ^{1}H NMR (400 MHz, $CDCl_3$, 20 °C, ppm): δ 7.55 – 7.50 (m, 2H, C$\underline{H}$), 7.44 (s, 1H, benzylic position), 7.40 (d, J = 8.2 Hz, 2H, C$\underline{H}$), 7.26 – 7.23 (m, 2H, C$\underline{H}$), 7.12 (d, J = 8.1 Hz, 2H, C$\underline{H}$), 1.32 (s, 12H, C$\underline{H_3}$). ^{13}C{^{1}H} NMR (101 MHz, $CDCl_3$, 20 °C, ppm) δ 143.7 (s, $\underline{Cq}$), 142.8 (s, $\underline{C}$H, benzylic position), 140.1(s, $\underline{Cq}$), 130.1 (s, $\underline{C}$H), 129.3 (s, $\underline{C}$H), 129.2 (q, J = 32.5 Hz, $\underline{C}CF_3$), 125.5 (q, J = 3.8 Hz, $\underline{C}$H), 125.2 (q, J = 3.8 Hz, $\underline{C}$H), 124.7 (q, J = 140.6 Hz, C$\underline{C}F_3$), 84.4 (s, $\underline{Cq}$ (Bpin)), 24.9 (s, $\underline{C}H_3$ (BPin)). ^{11}B{^{1}H} NMR (128 MHz, $CDCl_3$, 20 °C, ppm) δ 30.4. Spectroscopic data correspond to literature data.[20]

***(Z)*-2-(1,2-bis(4-ethylphenyl)vinyl)-4,4,5,5-tetramethyl-1,3,2-dioxaborolane (S8)**

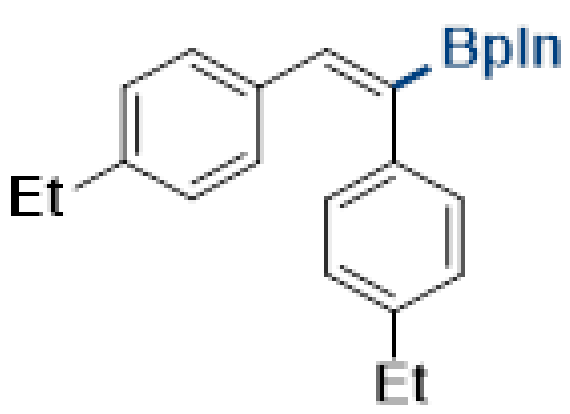

Conversion: >99% (E/Z: 1/99). Yield: 170.3 mg (94% isolated yield). Rt = 9.273 min (Method B), m/z= 362.24. ^{1}H NMR (400 MHz, $CDCl_3$, 20 °C, ppm):δ 7.32 (s, 1H, C$\underline{H}$), 7.13-7.09 (m, 4H, C$\underline{H}$), 7.04 – 6.94 (m, 4H, C$\underline{H}$), 2.66 (q, J = 7.6 Hz, 2H, C$\underline{H_2}$), 2.57 (q, J = 7.6 Hz, 2H, C$\underline{H_2}$), 1.32 (s, 12H, C$\underline{H_3}$), 1.29 – 1.24 (m, 3H, C$\underline{H_3}$), 1.19 (t, J = 7.6

Hz, 3H, C*H*$_3$).^{13}C{^{1}H} NMR (101 MHz, $CDCl_3$, 20 °C, ppm) δ 143.8 (s, *Cq*), 143.0(s, CH), 142.0 (s, *Cq*), 137.9 (s, *Cq*), 134.7 (s, *Cq*), 130.1 (s, CH), 128.8 (s, CH), 127.9 (s, CH), 83.8 (s, *Cq*),28.7 (s, CH_2) 24.9 (s, CH_3), 15.5 (s, CH_3), 15.3(s, CH_3).^{11}B {^{1}H} NMR (128 MHz, $CDCl_3$, 20 °C, ppm) δ 30.8 (s, B). Anal. Calcd for $C_{24}H_{31}BO_2$: C, 79.56; H, 8.62. Found: C, 79.50; H, 8.75.

***(Z)*-2-(1,2-bis(4-isopropylphenyl)vinyl)-4,4,5,5-tetramethyl-1,3,2-dioxaborolane (S9)**

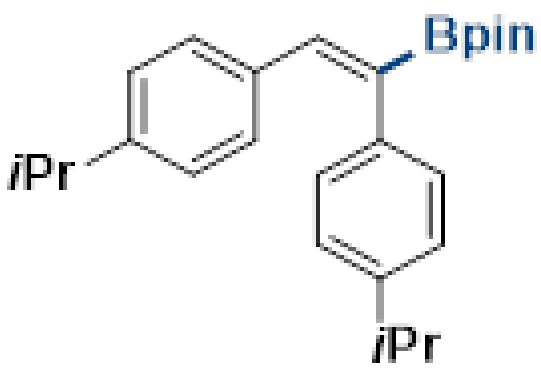

Conversion: >99% (E/Z: 1/99). Yield: 173.7 mg (94% isolated yield). Rt= 9.599min (Method B), m/z = 390.302. ^{1}H NMR (400 MHz, $CDCl_3$, 20 °C, ppm): δ 7.34 (s, 1H, benzylic position), 7.19 – 7.11 (m, 4H, C*H*), 7.06 – 6.96 (m, 4H, C*H*), 2.93 (pd, *J* = 6.9, 4.5 Hz, 2H, (C*H*)(CH_3)$_2$), 1.33 (s, 12H, C*H*$_3$ (BPin)), 1.31 – 1.27 (m, 12H, (CH)(C*H*$_3$)$_2$)). ^{13}C{^{1}H} NMR (101 MHz, $CDCl_3$, 20 °C, ppm) δ 148.5 (s, *Cq*), 146.7(s, *Cq*), 143.0 (s, CH, benzylic position), 138.0 (s, *Cq*), 134.8 (s, *Cq*), 130.2 (s, CH), 128.8 (s, CH), 126.4 (s, CH), 126.0 (s, CH), 83.7 (s, *Cq* (Bpin)), 33.9 (s, (CH)(CH_3)$_2$), 33.9 (s, (CH)(CH_3)$_2$), 24.9 (s, CH_3 (BPin)), 24.2 (s, (CH)(CH_3)$_2$), 24.0 (s, (CH)(CH_3)$_2$). ^{11}B{^{1}H} NMR (128 MHz, $CDCl_3$, 20 °C, ppm) δ 31.1 (s, B). Anal. Calcd for $C_{26}H_{35}BO_2$: C, 80.00; H, 9.05. Found: C, 79.87; H, 9.15.

***(Z)*-2-(1,2-bis(4-methoxyphenyl)vinyl)-4,4,5,5-tetramethyl-1,3,2-dioxaborolane (S10)**

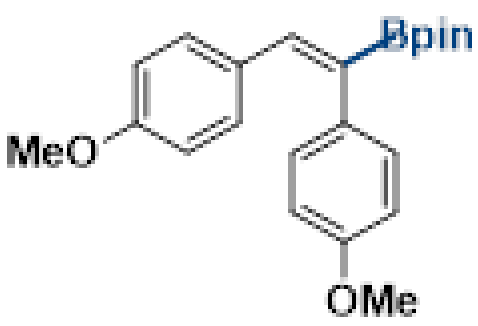

Conversion: 64% (GC-MS); NMR ratio: Educt/Product: 50/50. Rt = 8.436 min (Method B) m/z = 366.172. Could not be isolated in pure form. ^{1}H NMR (400 MHz, $CDCl_3$, 20 °C, ppm): δ 7.28 (s, 1H, benzylic position), 7.17 – 7.09 (m, 2H, C*H*), 7.08 – 7.03 (m, 2H, C*H*), 6.86 – 6.82 (m, 2H, C*H*), 6.70 – 6.64 (m, 2H, C*H*)), 3.81 (s, 3H, OC*H*$_3$), 3.75 (s, 3H, OC*H*$_3$), 1.31 (s, 12H, C*H*$_3$ (BPin). ^{13}C{^{1}H} NMR (101 MHz, $CDCl_3$, 20 °C, ppm): δ 159.1 (s, *Cq*), 158.1 (s, *Cq*), 142.5 (s, CH, benzylic position) , 133.1 (s, *Cq*) , 131.5 (s, CH), 130.1 (s, CH), 130.0 (s, *Cq*), 113.9 (s, CH), 113.4 (s, CH), 55.2(s, OCH_3) , 24.9 (s, CH_3 (BPin)). ^{11}B{^{1}H} NMR (128 MHz, $CDCl_3$, 20 °C, ppm) δ 31.1 (s, B). Spectroscopic data correspond to literature data.[21]

***(Z)*-2-(1,2-di-o-tolylvinyl)-4,4,5,5-tetramethyl-1,3,2-dioxaborolane (S11)**

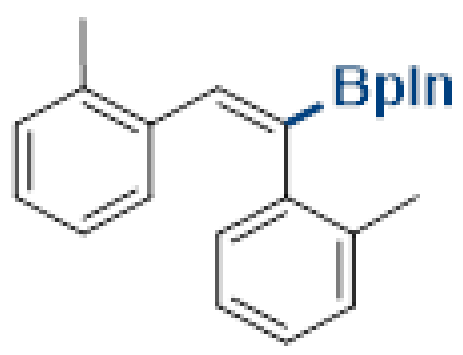

Conversion: 46% (E/Z: 1/99). Yield: 66.8 mg (40% isolated yield). Rt= 8.259min (Method B), m/z = 334.206. ^{1}H NMR (400 MHz, $CDCl_3$, 20 °C, ppm): δ 7.56 (s, 1H, C*H*), 7.13 – 7.05 (m, 3H, C*H*), 7.00 (td, *J* = 7.4, 1.6 Hz, 2H, C*H*), 6.86 (dd, *J* = 7.4, 1.4 Hz, 1H, C*H*), 6.76 (tt, *J* = 7.4, 0.9 Hz, 1H, C*H*), 6.64 – 6.58 (m, 1H, C*H*), 2.37 (s, 3H, C*H*$_3$), 2.14 (s, 3H, C*H*$_3$), 1.30 (s, 12H, C*H*$_3$). ^{13}C{^{1}H} NMR (101 MHz, $CDCl_3$, 20 °C, ppm): δ 142.5 (s, CH), 140.3 (s, *Cq*), 136.8 (s, *Cq*), 136.6 (s, *Cq*), 135.5 (s, *Cq*), 129.9 (s, CH), 129.9 (s, CH), 129.2 (s, CH), 129.1 (s, CH), 127.5 (s, CH), 126.4 (s, CH), 125.7 (s, CH), 125.2 (s, CH), 83.8 (s, *Cq*), 24.9 (s, CH_3), 20.1 (s, CH_3), 20.1 (s, CH_3). ^{11}B{^{1}H} NMR (128 MHz, $CDCl_3$, 20 °C, ppm) δ 31.1 (s, B). Spectroscopic data correspond to literature data.[18]

***(Z)*-2-(1,2-di(thiophen-3-yl)vinyl)-4,4,5,5-tetramethyl-1,3,2-dioxaborolane (S12)**

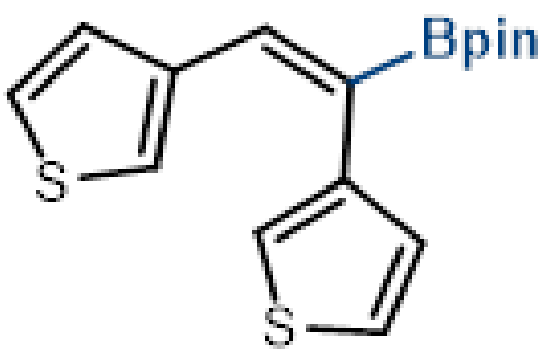

Conversion: 71% (E/Z: 1/99). Yield: 100.3mg (63% isolated yield). Rt= 8.259min (Method B), m/z = 318.259. ^{1}H NMR (400 MHz, CDCl$_3$, 20 °C, ppm): δ 7.34 (s, 1H, C*H*), 7.26 – 7.24 (m, 1H, C*H*), 7.11 (ddd, *J* = 3.0, 2.1, 1.3 Hz, 2H, C*H*), 7.07 (ddd, *J* = 5.1, 3.0, 0.5 Hz, 1H, C*H*), 6.92 (dd, *J* = 4.9, 1.3 Hz, 1H, C*H*), 6.72 – 6.69 (m, 1H, C*H*), 1.30 (s, 12H, C*H*$_3$). ^{13}C{^{1}H} NMR (101 MHz, CDCl$_3$, 20 °C, ppm): δ 140.5 (s, *C*$_q$), 139.0(s, *C*$_q$), 137.5 (s, *C*H), 128.8 (s, *C*H), 126.5 (s, *C*H), 124.6 (s, *C*H), 124.6 (s, *C*H), 122.2 (s, *C*H), 83.8 (s, *C*$_q$), 24.8 (s, *C*H$_3$). ^{11}B{^{1}H} NMR (128 MHz, CDCl$_3$, 20 °C, ppm) δ 30.6 (s, B). Spectroscopic data correspond to literature data.[19]

***(Z)*-4,4,5,5-tetramethyl-2-(oct-4-en-4-yl)-1,3,2-dioxaborolane (S13)**

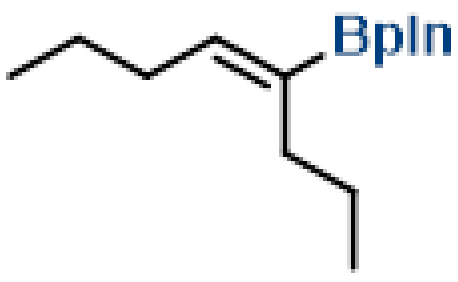

Conversion: 77% (E/Z: 14/86). Yield: 83.3 mg (70 % isolated yield). Rt= 13.983 min (Method A), m/z = 238.226. ^{1}H NMR (400 MHz, CDCl$_3$, 20 °C, ppm): δ 6.29 (t, *J* = 7.3 Hz, 1H, C*H*), 2.19 – 2.03 (m, 4H, C*H*$_2$), 1.46 – 1.29 (m, 4H, C*H*$_2$), 1.24 (s, 12H, C*H*$_3$), 0.98 – 0.90 (m, 3H, C*H*$_3$), 0.90 – 0.83 (m, 4H, C*H*$_3$). ^{13}C{^{1}H} NMR (101 MHz, CDCl$_3$, 20 °C, ppm): δ 146.1 (s, *C*H), 83.0 (s, *C*$_q$), 30.8 (s, *C*H$_2$), 30.7 (s, *C*H$_2$), 24.9 (s, *C*H$_3$), 23.4 (s, *C*H$_2$), 22.5 (s, *C*H$_2$), 14.2 (s, *C*H$_3$), 14.2 (s, *C*H$_3$). Spectroscopic data correspond to literature data.[18]

***(E)*-4,4,5,5-tetramethyl-2-styryl-1,3,2-dioxaborolane (S14)**

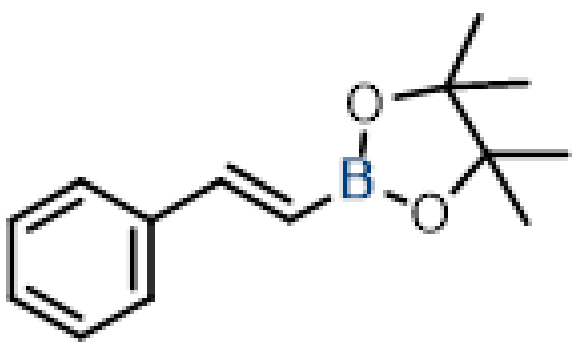

Conversion: 45% (E/Z: 97/3). Isolated yield: 44.4 mg (38%). Rt = 17.071 min (Method A), m/z = 230.179. ^{1}H NMR (400 MHz, CDCl$_3$, 20 °C, ppm): δ 7.55 – 7.46 (m, 2H,C*H*), 7.40 (d, *J* = 18.5 Hz, 1H, C*H*), 7.36 – 7.28 (m, 3H, C*H*), 6.17 (d, *J* = 18.4 Hz, 1H, C*H*), 1.31 (s, 12H, C*H*$_3$). ^{13}C{^{1}H} NMR (101 MHz, CDCl$_3$, 20 °C, ppm): δ 149.5 (s, *C*H), 130.8 (s, *C*$_q$), 128.9 (s, *C*H), 128.6 (s, *C*H), 127.1 (s, *C*H), 83.4 (s, *C*$_q$), 24.8 (s, *C*H$_3$). ^{11}B{^{1}H} NMR (128 MHz, CDCl$_3$, 20 °C, ppm): δ 30.4 (s, B). Spectroscopic data correspond to literature data. [22]

***(E)*-2-(2-cyclohexylvinyl)-4,4,5,5-tetramethyl-1,3,2-dioxaborolane (S15)**

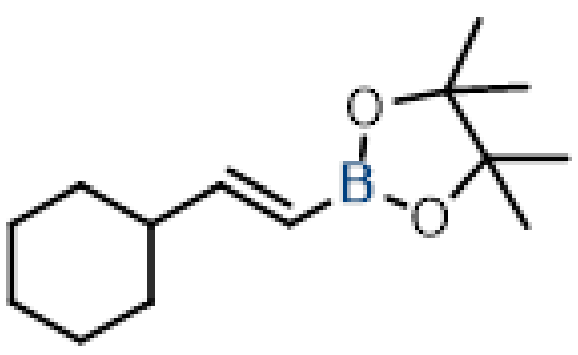

Conversion >99% (E/Z: 97/3). Isolated yield: 89.2 mg (70%). Rt = 10.187 min (Method A), m/z = 236.224. ^{1}H NMR (400 MHz, CDCl$_3$, 20 °C, ppm): δ 6.51 (dd, *J* = 18.2, 6.2 Hz, 1H, C*H*), 5.30 (dd, *J* = 18.1, 1.5 Hz, 1H, C*H*), 1.93 (dtd, *J* = 7.7, 4.1, 2.3 Hz, 1H, C*H*), 1.73 – 1.62 (m, 6H, C*H*$_2$), 1.57 (dtt, *J* = 12.8, 3.3, 1.6 Hz, 2H, C*H*$_2$), 1.19 (s, 12H, C*H*$_3$), 1.16 – 0.96 (m, 2H C*H*$_2$). ^{13}C{^{1}H} NMR (101 MHz, CDCl$_3$, 20 °C, ppm): δ 159.9 (s, *C*H), 83.1 (s, *C*$_q$), 32.1 (s, *C*H$_2$), 26.3 (s, *C*H$_2$), 24.90 (s, *C*H$_3$), 21.1 (s, *C*H). ^{11}B{^{1}H} NMR (128 MHz, CDCl$_3$, 20 °C, ppm): δ 29.7(s, B). Spectroscopic data correspond to literature data.[23]

***(E)*-2-(6-chlorohex-1-en-1-yl)-4,4,5,5-tetramethyl-1,3,2-dioxaborolane (S16)**

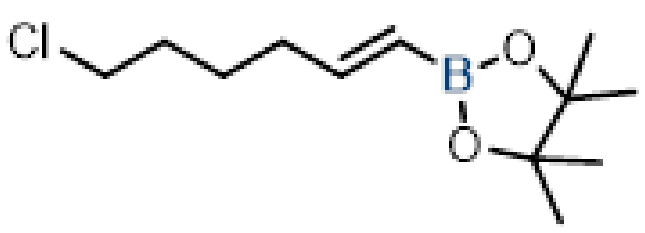

Conversion95% (E/Z: 96/4). Isolated yield: 89.2 mg (72%). Rt = 10.143 min (Method A), m/z = 244.155. ^{1}H NMR (400 MHz, CDCl$_3$, 20 °C, ppm): δ 6.58 (dt, *J* = 18.0, 6.5 Hz, 1H, C*H*), 5.42 (dt,

J = 17.9, 1.6 Hz, 1H, CH)), 3.50 (t, J = 6.7 Hz, 2H, CH_2-Cl)), 2.16 (tdd, J = 7.5, 6.3, 1.6 Hz, 2H, CH_2), 1.81 – 1.71 (m, 2H, CH_2), 1.61 – 1.50 (m, 2H, CH_2), 1.24 (s, 12H, CH_3). $^{13}C\{^{1}H\}$ NMR (101 MHz, $CDCl_3$, 20 °C, ppm): δ 159.9 (s, CH-B), 153.5 (s, CH), 83.1 (s, C_q), 44.9 (s, CH_2), 34.9(s, CH_2), 32.1(s, CH_2), 25.5(s, CH_2), 24.9 (s, CH_3). $^{11}B\{^{1}H\}$ NMR (128 MHz, $CDCl_3$, 20 °C, ppm): δ 29.7(s, B). Spectroscopic data corresponds to literature data.[24]

***(E)*-4,4,5,5-tetramethyl-2-(5-methylhex-1-en-1-yl)-1,3,2-dioxaborolane (S17)**

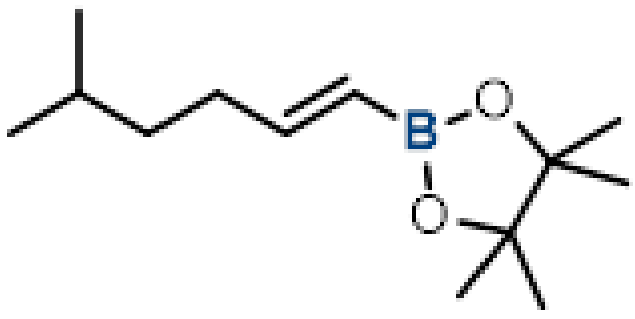

Conversion: 98% (E/Z: 97/3). Isolated yield: 77.4 mg (68%). Rt = 15.339 min (Method A), m/z = 224.244. ^{1}H NMR (400 MHz, $CDCl_3$, 20 °C, ppm): δ 6.62 (dt, J = 18.0, 6.5 Hz, 1H, CH), 5.42 (dt, J = 17.9, 1.6 Hz, 1H, CH), 2.15 (dtd, J = 9.4, 6.5, 1.6 Hz, 2H, CH_2), 1.55 (dp, J = 13.3, 6.7 Hz, 1H CH), 1.34 – 1.27 (m, 2H, CH_2), 1.26 (s, 12H, CH_3), 0.86 (d, J = 6.6 Hz, 6H, CH_3). $^{13}C\{^{1}H\}$ NMR (101 MHz, $CDCl_3$, 20 °C, ppm): δ 155.1 (s, CH), 83.1 ((s, C_q), 37.5 (s, CH_2), 33.8 (s, CH_2), 27.6 (s, CH), 24.9 (s, CH_3), 22.6 (s, CH_3). $^{11}B\{^{1}H\}$ NMR (128 MHz, $CDCl_3$, 20 °C, ppm): δ 30.1 (s, B). Spectroscopic data correspond to literature data.[23]

***(E)*-4,4,5,5-tetramethyl-2-(oct-1-en-1-yl)-1,3,2-dioxaborolane (S18)**

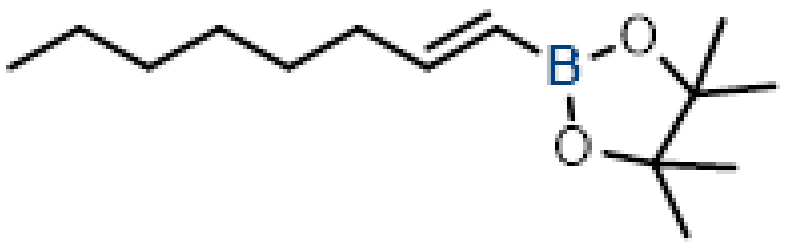

Conversion: 73% (E/Z: 99/1). Isolated yield: 78.6 mg (65%). Rt = 13.908 min (Method A), m/z = 238.282. ^{1}H NMR (400 MHz, $CDCl_3$, 20 °C, ppm): δ 6.63 (dt, J = 17.9, 6.4 Hz, 1H, CH), 5.42 (dt, J = 17.9, 1.6 Hz, 1H, CH), 2.19 – 2.09 (m, 3H,), 1.45 – 1.35 (m, 3H, CH_2), 1.26 (s, 15H, CH_2), 0.91 – 0.84 (m, 5H, CH_2). $^{13}C\{^{1}H\}$ NMR (101 MHz, $CDCl_3$, 20 °C, ppm): δ 155.0 (s, CH), 83.1 (s, C_q), 36.0 (s, CH_2), 31.9(s, CH_2), 29.1(s, CH_2), 28.4 (s, CH_2), 24.9 (s, CH_2), 22.8 (s, CH_3), 14.2 (s, CH_3). $^{11}B\{^{1}H\}$ NMR (128 MHz, $CDCl_3$, 20 °C, ppm): δ 30.0 (s, B). Spectroscopic data correspond to literature data.[23]

References

(1) Perrin, D. D.; Armarego, W. L. F. *Purification of Laboratory Chemicals*, 3rd ed.; Pergamon: New York, **1988**.

(2) Eder, W.; Himmelbauer, D.; Stöger, B.; Veiros, L. F.; Pignitter, M.; Kirchner, K. Manganese and Iron PCP Pincer Complexes - The Influence of the Linkers on Structure and Reactivity *Dalton Trans.* **2021**, *50*, 13915-13924.

(3) Käfer, M.; Gstöttner, S.; Stöger, B.; Dupé, A.; Veiros, L. F.; Kirchner, K. Synthesis, Characterization and Reactivity of Alkyl Iron(II) PCP Pincer Complexes *Organometallics* **2026**, in press doi.org/10.1021/acs.organomet.6c00256.

(4) Zhou, B.; Wu, Z.; Ma, D.; Ji, X.; Zhang, Y.; Synthesis of Indoles through Palladium-Catalyzed Three-Component Reaction of Aryl Iodides, Alkynes, and Diaziridinone *Org. Lett.* **2018**, *20*, 6440-6443.

(5) Park, K.; Bae, G.; Moon, J.; Choe, J.; Song, K.; Lee, S.; Synthesis of Symmetrical and Unsymmetrical Diarylalkynes from Propiolic Acid Using Palladium-Catalyzed Decarboxylative Coupling *J. Org. Chem.* **2010**, *75*, 6244-6251.

(5) GAUSSIAN 09, Revision A.01, Frisch, M. J.; Trucks, G. W.; Schlegel, H. B.; Scuseria, G. E.; Robb, M. A.; Cheeseman, J. R.; Scalmani, G.; Barone, V.; Mennucci, B.; Petersson, G. A.; Nakatsuji, H.; Caricato, M.; Li, X.; Hratchian, H. P.; Izmaylov, A. F.; Bloino, J.; Zheng, G.; Sonnenberg, J. L.; Hada, M.; Ehara, M.; Toyota, K.; Fukuda, R.; Hasegawa, J.; Ishida, M.; Nakajima, T.; Honda, Y.; Kitao, O.; Nakai, H.; Vreven, T.; Montgomery, Jr., J. A.; Peralta, J. E.; Ogliaro, F.; Bearpark, M.; Heyd, J. J.; Brothers, E.; Kudin, K. N.; Staroverov, V. N.; Kobayashi, R.; Normand, J.; Raghavachari, K.; Rendell, A.; Burant, J. C.; Iyengar, S. S.; Tomasi, J.; Cossi, M.; Rega, N.; Millam, J. M.; Klene, M.; Knox, J. E.; Cross, J. B.; Bakken, V.; Adamo, C.; Jaramillo, J.; Gomperts, R.; Stratmann, R. E.; Yazyev, O.; Austin, A. J.; Cammi, R.; Pomelli, C.; Ochterski, J. W.; Martin, R. L.; Morokuma, K.; Zakrzewski, V. G.; Voth, G. A.; Salvador, P.; Dannenberg, J. J.; Dapprich, S.; Daniels, A. D.; Farkas, Ö.; Foresman, J. B.; Ortiz, J. V.; Cioslowski, J.; Fox, D. J. Gaussian, Inc., Wallingford CT, **2009**.

(6) Hehre, W. J.; Radom, L.; Schleyer, P. v.R.; Pople, J. A. *Ab Initio Molecular Orbital Theory*, John Wiley & Sons, NY, **1986**.

(7) Parr, R. G.; Yang, W. *Density Functional Theory of Atoms and Molecules*; Oxford University Press: New York, **1989**.

(8) (a) Perdew, J. P.; Burke, K.; Ernzerhof, M. Generalized Gradient Approximation Made Simple *Phys. Rev. Lett.* **1996**, *77*, 3865-3868; (b) Perdew, J. P.; Burke, K.; Ernzerhof, M. Generalized Gradient Approximation Made Simple *Phys. Rev. Lett.* **1997**, *78*, 1396-1396. (c) Perdew, J. P. Density-functional approximation for the correlation energy of the inhomogeneous electron gas *Phys. Rev. B* **1986**, *33*, 8822-8824.

(9) (a) Haeusermann, U.; Dolg, M.; Stoll, H.; Preuss, H.; Schwerdtfeger, P.; Pitzer, R. M. Accuracy of energy-adjusted quasirelativistic ab initio pseudopotentials *Mol. Phys.* **1993**, *78*, 1211-1224. (b) Kuechle, W.; Dolg, M.; Stoll, H.; Preuss, H. Energy-adjusted pseudopotentials for the actinides. Parameter sets and test calculations for thorium and thorium monoxide *J. Chem. Phys.* **1994**, *100*, 7535-7542. (c) Leininger, T.; Nicklass, A.; Stoll, H.; Dolg, M.; Schwerdtfeger, P. The accuracy of the pseudopotential approximation. II. A comparison of various core sizes for indium pseudopotentials in calculations for spectroscopic constants of InH, InF, and InCl *J. Chem. Phys.* **1996**, *105*, 1052-1059.

(10) (a) Ditchfield, R.; Hehre, W. J.; Pople, J. A. Self-Consistent Molecular-Orbital Methods. IX. An Extended Gaussian-Type Basis for Molecular-Orbital Studies of Organic Molecules *J. Chem. Phys.* **1971**, *54*, 724-728. (b) Hehre, W. J.; Ditchfield, R.; Pople, J. A. Self-Consistent Molecular Orbital Methods. 12. Further extensions of Gaussian-type basis sets for use in molecular-orbital studies of organic-molecules *J. Chem. Phys.* **1972**, *56*, 2257-2261. (c) Hariharan, P. C.; Pople, J. A. Accuracy of AH equilibrium geometries by single determinant molecular-orbital theory *Mol. Phys.* **1974**, *27*, 209-214. (d) Gordon, M. S. The isomers of silacyclopropane *Chem. Phys. Lett.* **1980**, *76*, 163-168. (e) Hariharan, P. C.; Pople, J. A. Influence of polarization functions on molecular-orbital hydrogenation energies *Theor. Chim. Acta* **1973**, *28*, 213-222.

(11) (a) Peng, C.; Ayala, P. Y.; Schlegel, H. B.; Frisch, M. J. Using redundant internal coordinates to optimize equilibrium geometries and transition states *J. Comp. Chem.* **1996**, *17*, 49-56. (b) Peng, C.; Schlegel, H. B. Combining Synchronous Transit and Quasi-Newton Methods for Finding Transition States *Israel J. Chem.* **1993**, *33*, 449-454.

(12) (a) McClean, A. D.; Chandler, G. S. Contracted Gaussian basis sets for molecular calculations. I. Second row atoms, *Z*=11-18 *J. Chem. Phys.* **1980**, *72*, 5639-5648. (b) Krishnan, R.; Binkley, J. S.; Seeger, R.; Pople, J. A. Self-consistent molecular orbital methods. XX. A basis set for correlated wave functions *J. Chem. Phys.* **1980**, *72*, 650-654. (c) Wachters, A. J. H. Gaussian Basis Set for Molecular Wavefunctions Containing Third-Row Atoms *J. Chem. Phys.* **1970**, *52*, 1033-1036. (d) Hay, P. J. Gaussian basis sets for molecular calculations - representation of 3D orbitals in transition-metal atoms *J. Chem. Phys.* **1977**, *66*, 4377-4384. (e) Raghavachari, K.; Trucks, G. W. Highly correlated systems: Excitation energies of first row transition metals Sc-Cu *J. Chem. Phys.* **1989**, *91*, 1062-1065. (f) Binning Jr., R. C.; Curtiss, L. A. Compact contracted basis-sets for 3rd-row atoms - Ga-Kr *J. Comp. Chem.* **1990**, *11*, 1206-1216. (g) McGrath, M. P.; Radom, L. Extension of Gaussian-1 (G1) theory to bromine-containing molecules *J. Chem. Phys.* **1991**, *94*, 511-516. (h) Curtiss, L. A.; McGrath, M. P.; Blaudeau, J.-P.; Davis, N. E.; Binning Jr., R. C.; Radom, L. Extension of Gaussian-2 theory to molecules containing third-row atoms Ga-Kr *J. Chem. Phys.*, **1995**, *103*, 6104-6113. (i) Clark, T.; Chandrasekhar, J.; Spitznagel, G. W.; Schleyer, P. v. R. Efficient diffuse function-augmented basis-sets for anion calculations. 3. The 3-21+G basis set for 1st-row elements, Li-F *J. Comp. Chem.* **1983**, *4*, 294-301. (j) Frisch, M. J.; Pople, J. A.; Binkley, J. S. Self-Consistent Molecular Orbital Methods. 25. Supplementary Functions for Gaussian Basis Sets *J. Chem. Phys.* **1984**, *80*, 3265-3269.

(13) (a) Cancès, M. T.; Mennucci, B.; Tomasi, J. A new integral equation formalism for the polarizable continuum model: Theoretical background and applications to isotropic and anistropic dielectrics *J. Chem. Phys.* **1997**, *107*, 3032-3041. (b) Cossi, M.; Barone, V.; Mennucci, B.; Tomasi, J. Ab initio study of ionic solutions by a polarizable continuum dielectric model *Chem. Phys. Lett.* **1998**, *286*, 253-260. (c) Mennucci, B.; Tomasi, J. Continuum solvation models: A new approach to the problem of solute's charge distribution and cavity boundaries *J. Chem. Phys.* **1997**, *106*, 5151-5158. (d) Tomasi, J.; Mennucci, B.; Cammi, R. Quantum mechanical continuum solvation models *Chem. Rev.* **2005**, *105*, 2999-3094.

(14) Marenich, A. V.; Cramer, C. J.; Truhlar, D. G. Universal solvation model based on solute electron density and a continuum model of the solvent defined by the bulk dielectric constant and atomic surface tensions *J. Phys. Chem. B*, **2009**, *113*, 6378-6396.

(15) Grimme, S.; Antony, J.; Ehrlich, S.; Krieg, H. A consistent and accurate ab initio parameterization of density functional dispersion correction (DFT-D) for the 94 elements H-Pu *J. Chem. Phys.* **2010**, *132*, 154104.

(16) (a) Becke, A. D.; Johnson, E. R. A density-functional model of the dispersion interaction *J. Chem. Phys.* **2005**, *122*, 154101. (b) Johnson, E. R.; Becke, A. D. A post-Hartree-Fock model of intermolecular interactions *J. Chem. Phys.* **2005**, *123*, 24101. (c) Johnson, E. R.; Becke, A. D. A post-Hartree-Fock model of intermolecular interactions: Inclusion of higher-order corrections *J. Chem. Phys.* **2006**, *124*, 174104.

(17) M. Espinal-Viguri, C. R. Woof, R. L. Webster, Iron-Catalyzed Hydroboration: Unlocking Reactivity through Ligand Modulation *Chem. Eur. J.* **2016**, *22*, 11605

(18) Rami F., Bächtle F., Plietker B. Hydroboration of internal alkynes catalyzed by $FeH(CO)(NO)(PPh_3)_2$: a case of boron-source controlled regioselectivity *Catal. Sci. Technol.*, **2020**, *10*, 1492-1497.

(19) A. Brzozowska, V. Zubar, R.-C. Ganardi, M. Rueping Chemoselective hydroboration of propargylic alcohols and amines using a manganese (II) catalyst *Org. Lett.* **2020**, *22*, 3765-3769.

(20) Zhou, X., Xu, Y. & Dong, G. Deacylation-aided C–H alkylative annulation through C–C cleavage of unstrained ketones. *Nat Catal.* **2021** *4*, 703–710.

(21) S. Wang, Ch. Chen, X. Zeng *Chin. J. Org. Chem.* **2023**, *43*, 2447-2453.

(22) S. Garhwal, N.Fridman, and G. de Ruiter Z-Selective Alkyne Functionalization Catalyzed by a trans-Dihydride N-Heterocyclic Carbene (NHC) Iron Complex *Inorg.Chem.,* **2020,** *59*, 13817-13821.

(23) D. P. Zobernig, B. Stöger, L. F. Veiros, and K. Kirchner Hydroboration of Terminal Alkynes Catalyzed by a Mn (I) Alkyl PCP Pincer Complex Following Two Diverging Pathways *ACS Catal.,* **2024,** *14*, 12385-12391.

(24) R. B. Pawar, M. H. Karmur, B. Punji Ligand-free $MnBr_2$-Catalyzed Chemo- and Stereoselective Hydroboration of Terminal Alkynes *Chem. Asian J.* **2024**, 19, e202400158.

NMR Spectra of New Organic Products

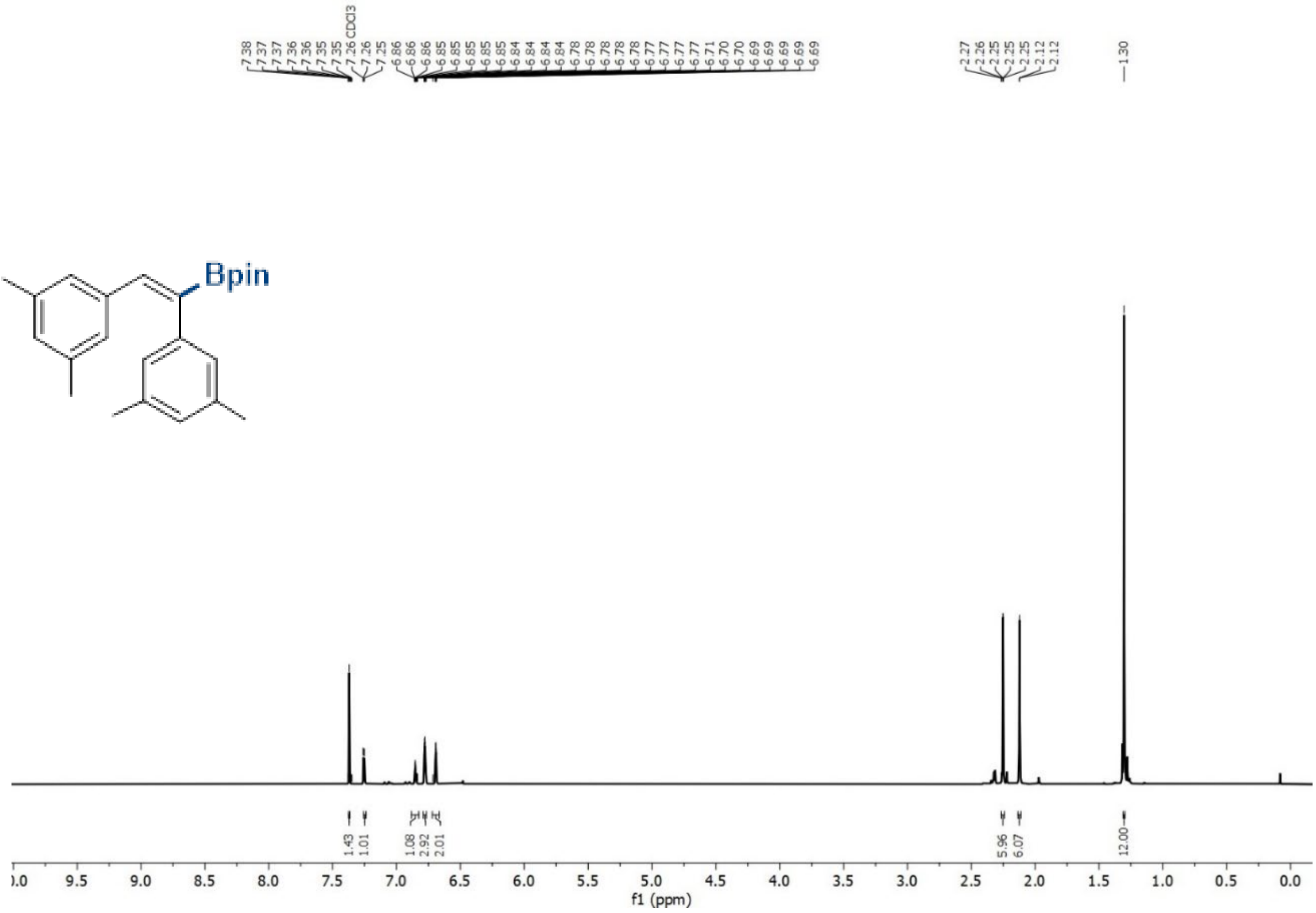

Figure S3. ^{1}H-NMR($CDCl_3$) of *(Z)*-2-(1,2-bis(3,5-dimethylphenyl)vinyl)-4,4,5,5-tetramethyl-1,3,2-dioxaborolane **(S3)**

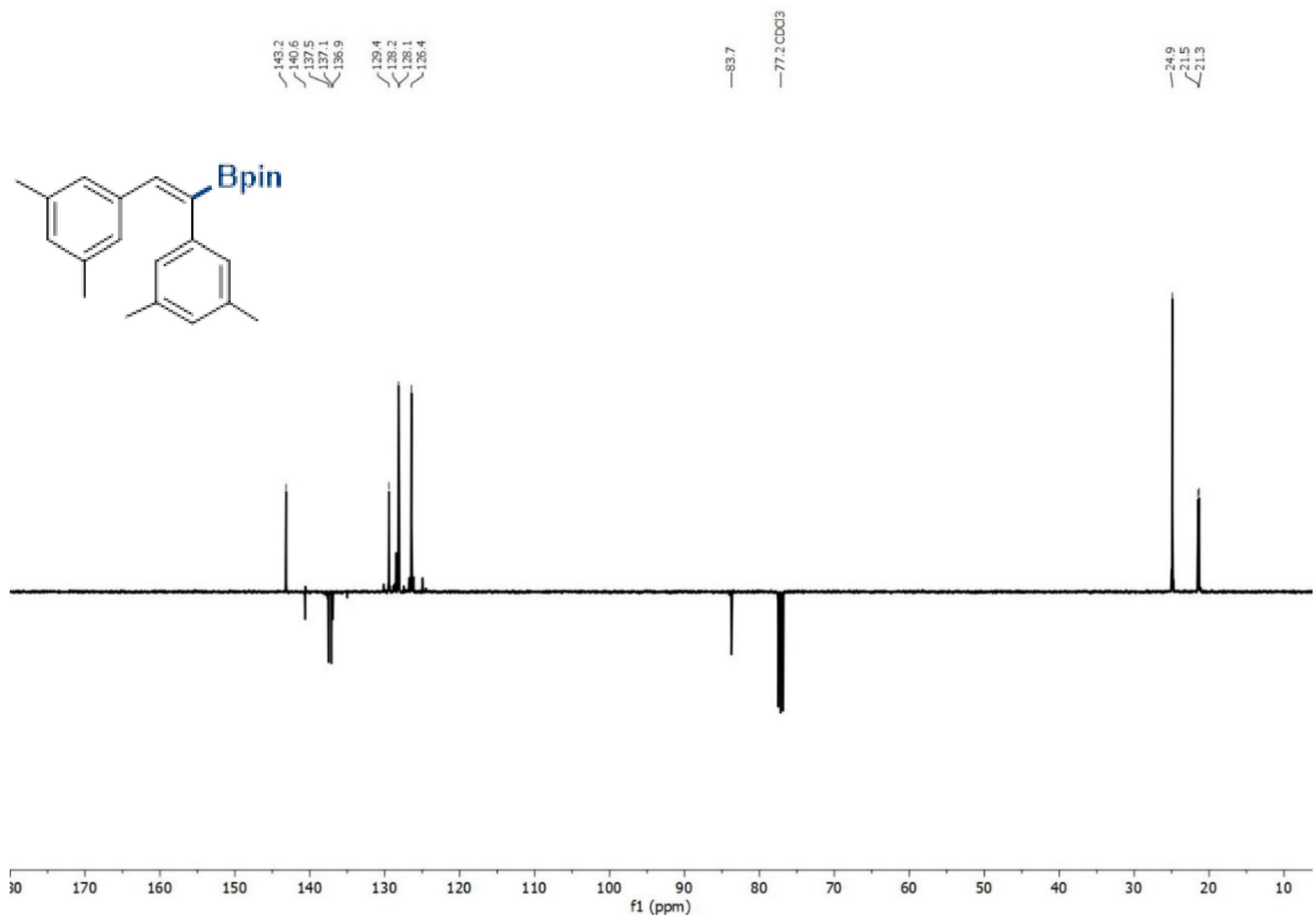

Figure S4. $^{13}C\{^{1}H\}$-NMR ($CDCl_3$) of *(Z)*-2-(1,2-bis(3,5-dimethylphenyl)vinyl)-4,4,5,5-tetramethyl-1,3,2-dioxaborolane **(S3)**

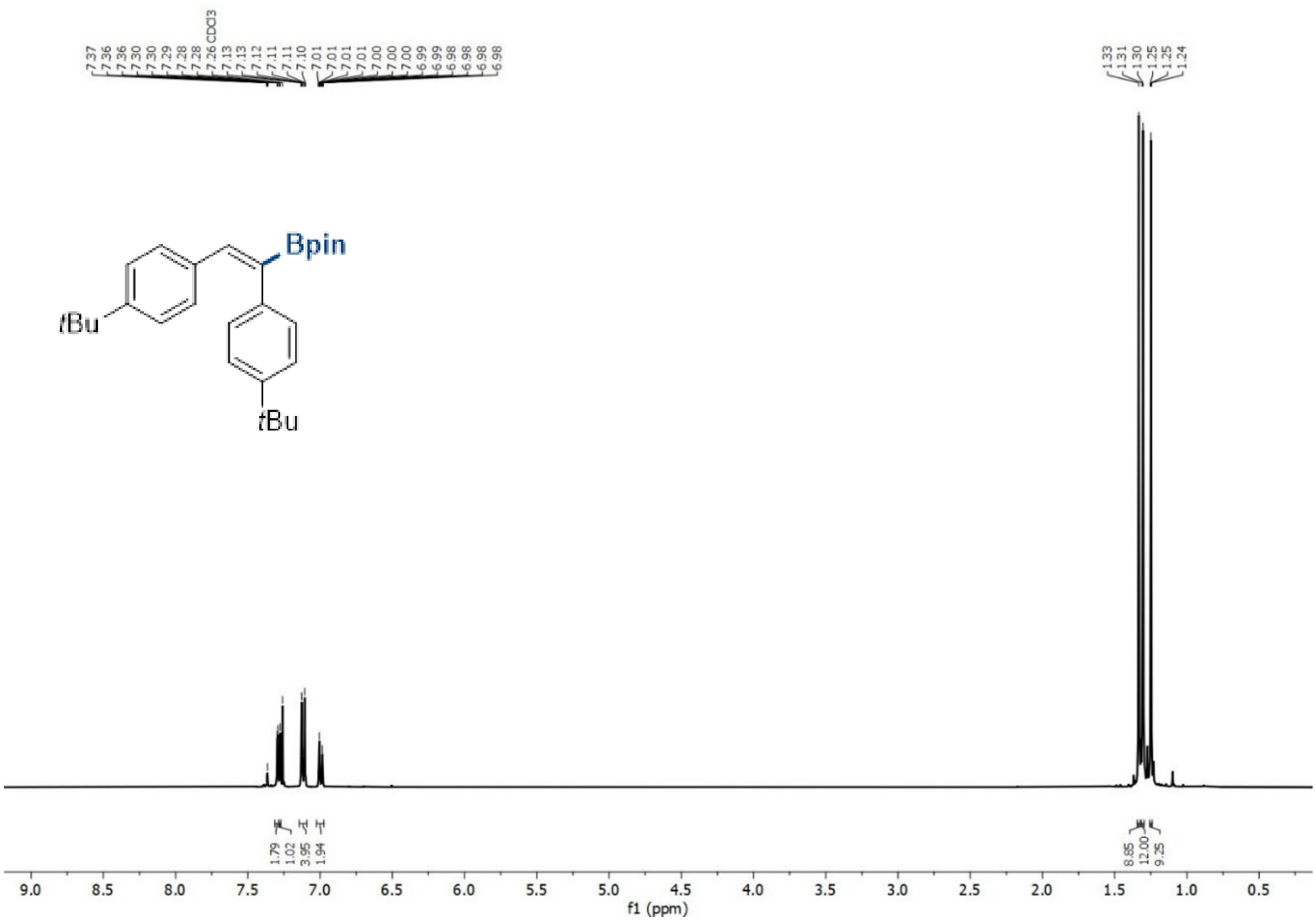

Figure S5. ^{1}H-NMR ($CDCl_3$) of *(Z)*-2-(1,2-bis(3,5-dimethylphenyl)vinyl)-4,4,5,5-tetramethyl-1,3,2-dioxaborolane (**S4**)

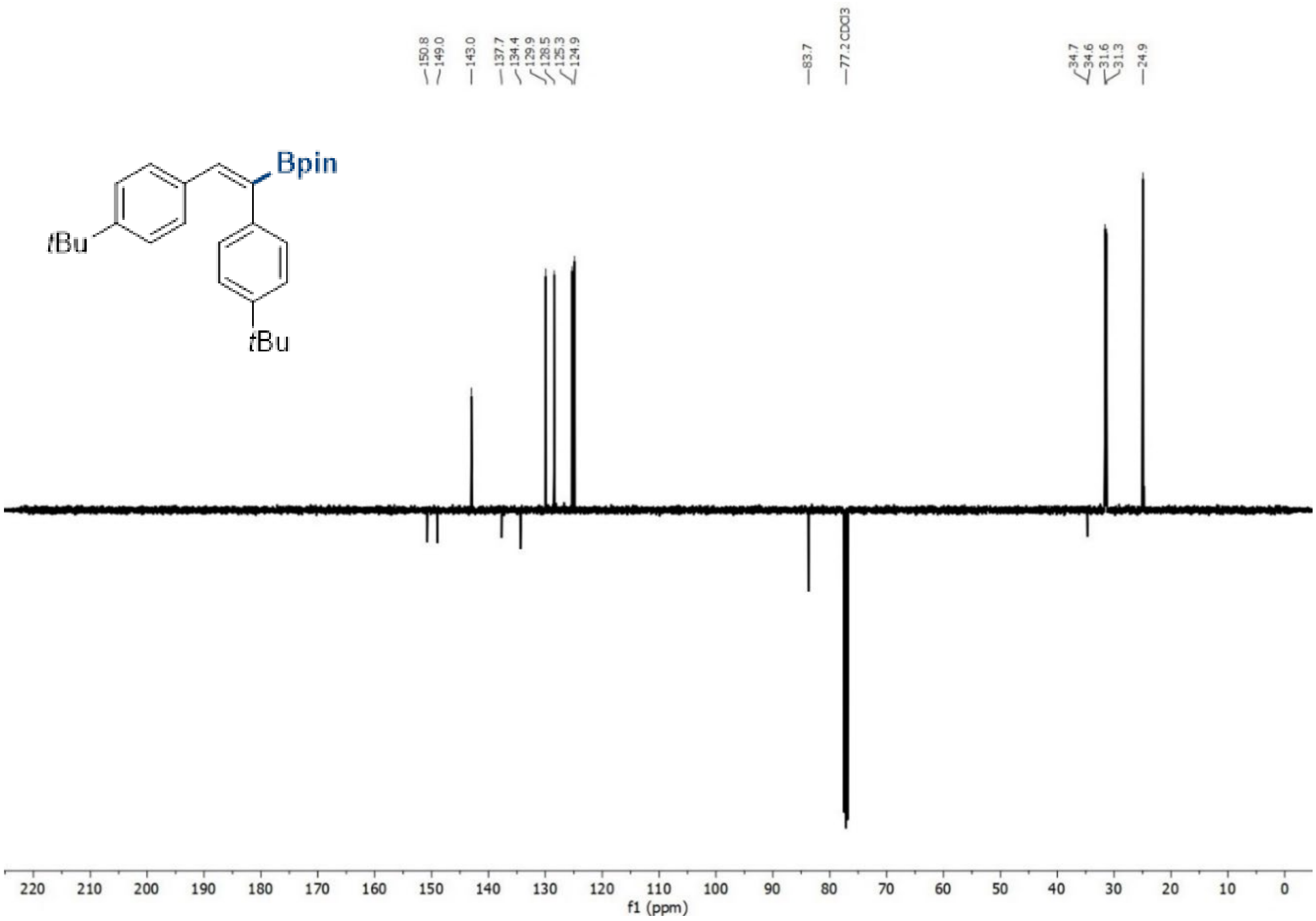

Figure S6. $^{13}C\{^{1}H\}$-NMR($CDCl_3$) of *(Z)*-2-(1,2-bis(3,5-dimethylphenyl)vinyl)-4,4,5,5-tetramethyl-1,3,2-dioxaborolane (**S4**)

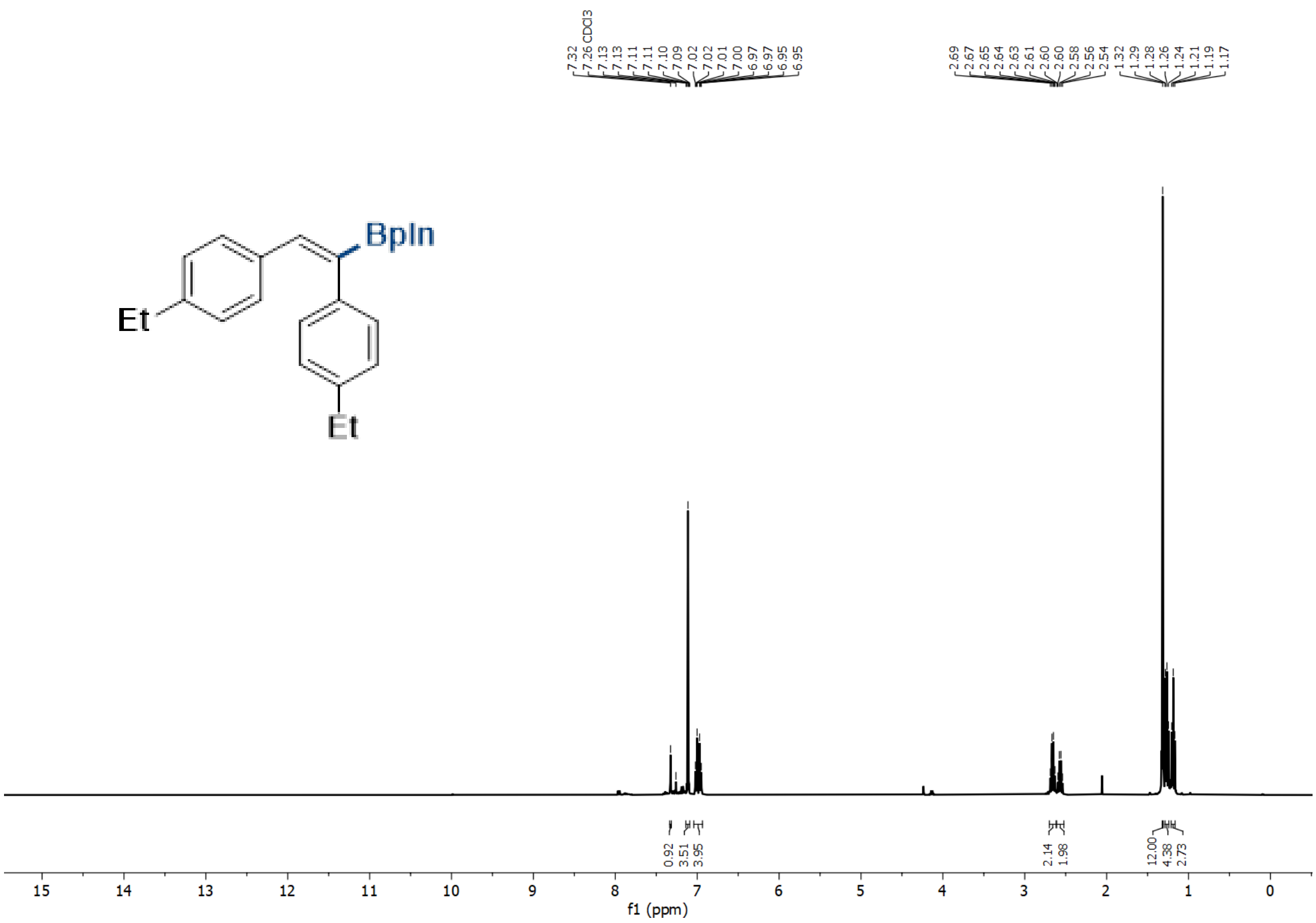

Figure S7. ^{1}H-NMR ($CDCl_3$) of *(Z)*-2-(1,2-bis(4-ethylphenyl)vinyl)-4,4,5,5-tetramethyl-1,3,2-dioxaborolane (**S8**)

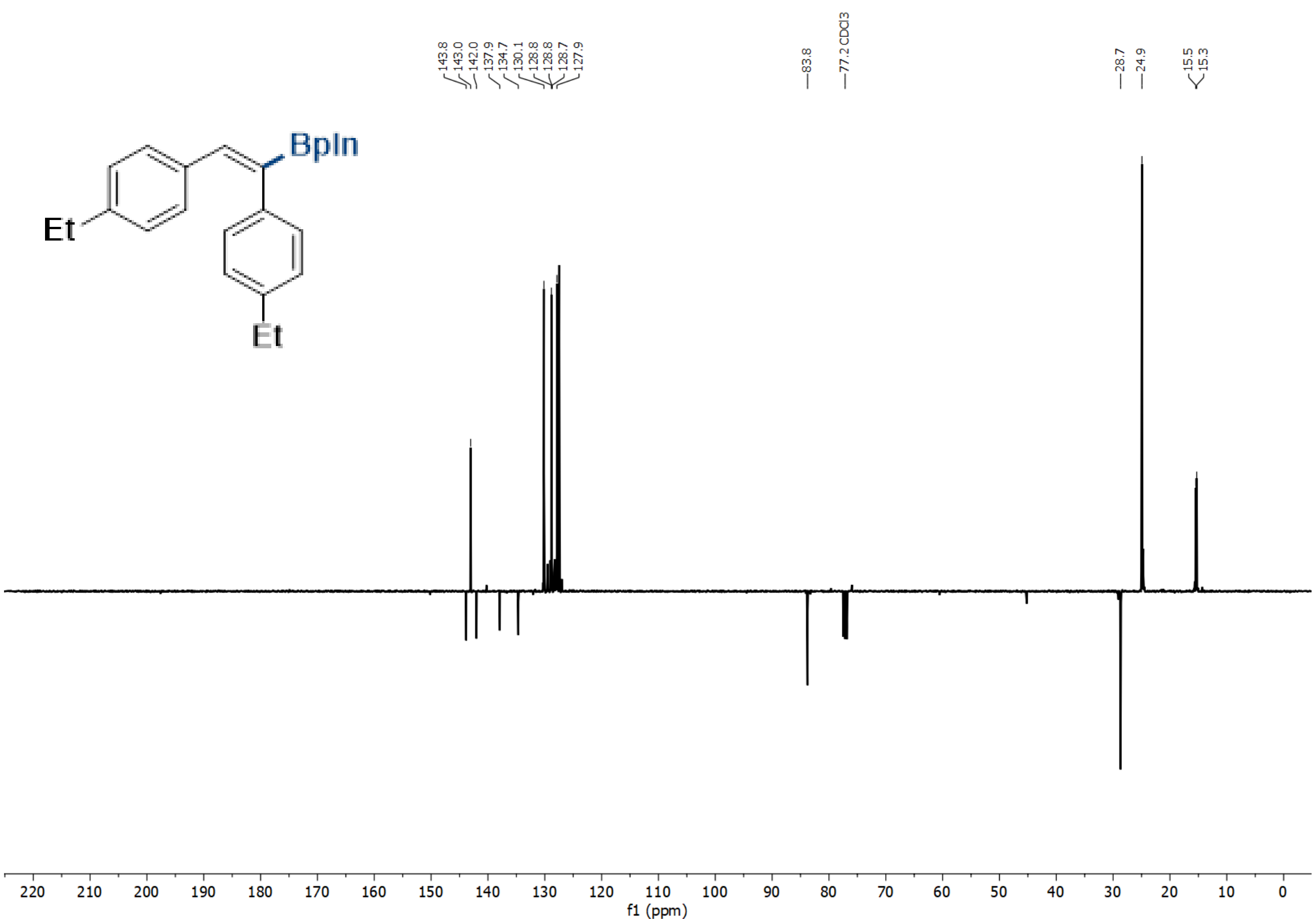

Figure S8. $^{13}C\{^{1}H\}$-NMR ($CDCl_3$) of *(Z)*-2-(1,2-bis(4-ethylphenyl)vinyl)-4,4,5,5-tetramethyl-1,3,2-dioxaborolane (**S8**)

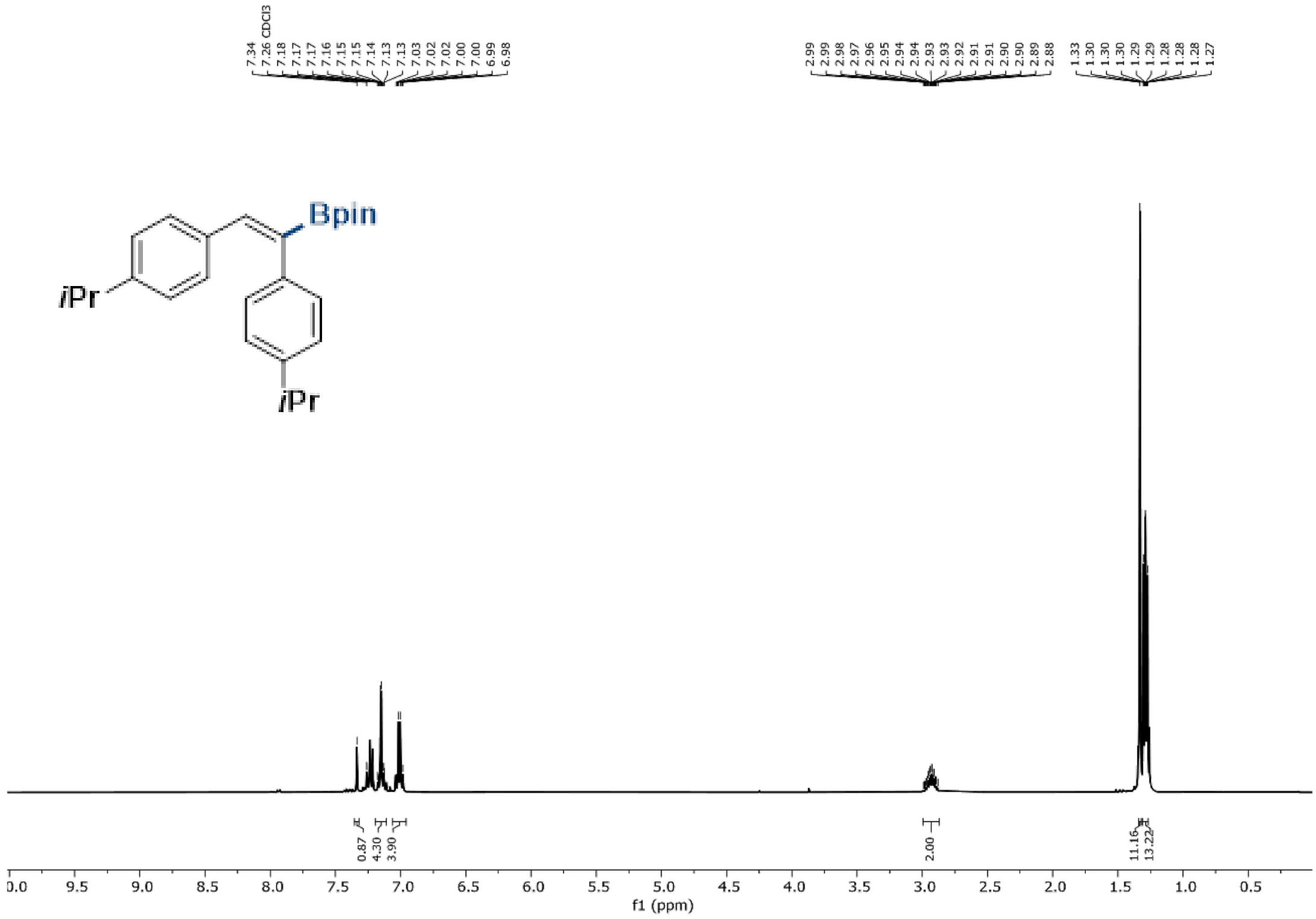

Figure S9. ^{1}H-NMR($CDCl_3$) of *(Z)*-2-(1,2-bis(4-isopropylphenyl)vinyl)-4,4,5,5-tetramethyl-1,3,2-dioxaborolane (**S9**)

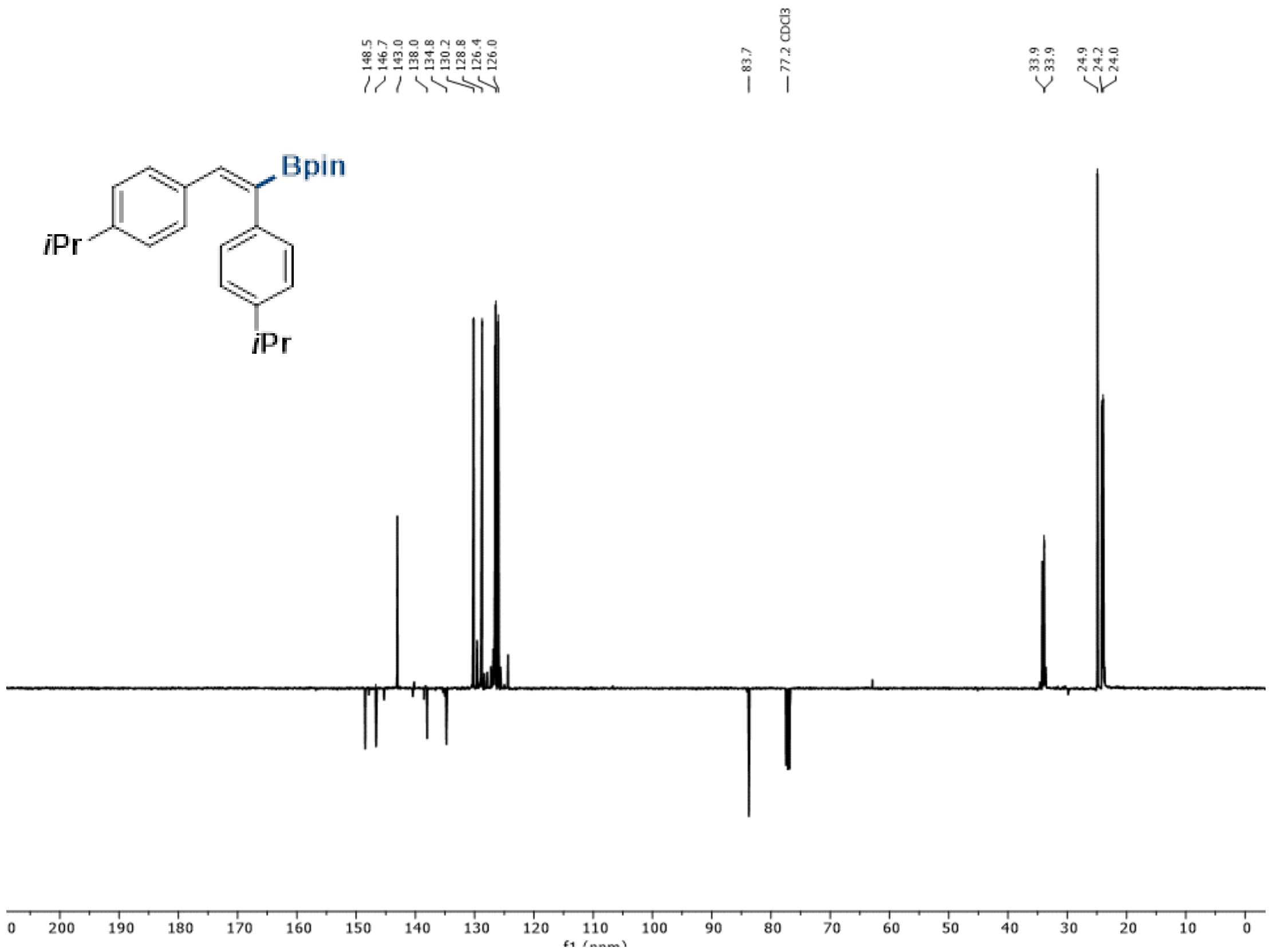

Figure S10. $^{13}C\{^{1}H\}$-NMR ($CDCl_3$) of *(Z)*-2-(1,2-bis(4-isopropylphenyl)vinyl)-4,4,5,5-tetramethyl-1,3,2-dioxaborolane (**S9**)
